# Identification of QTL for UV-Protective Eye Area Pigmentation in Cattle by Progeny Phenotyping and Genome-Wide Association Analysis

**DOI:** 10.1371/journal.pone.0036346

**Published:** 2012-05-02

**Authors:** Hubert Pausch, Xiaolong Wang, Simone Jung, Dieter Krogmeier, Christian Edel, Reiner Emmerling, Kay-Uwe Götz, Ruedi Fries

**Affiliations:** 1 Lehrstuhl fuer Tierzucht, Technische Universitaet Muenchen, Freising, Germany; 2 Institut fuer Tierzucht, Bayerische Landesanstalt für Landwirtschaft, Poing, Germany; University of Queensland, Australia

## Abstract

Pigmentation patterns allow for the differentiation of cattle breeds. A dominantly inherited white head is characteristic for animals of the Fleckvieh (FV) breed. However, a minority of the FV animals exhibits peculiar pigmentation surrounding the eyes (ambilateral circumocular pigmentation, ACOP). In areas where animals are exposed to increased solar ultraviolet radiation, ACOP is associated with a reduced susceptibility to bovine ocular squamous cell carcinoma (BOSCC, eye cancer). Eye cancer is the most prevalent malignant tumour affecting cattle. Selection for animals with ACOP rapidly reduces the incidence of BOSCC. To identify quantitative trait loci (QTL) underlying ACOP, we performed a genome-wide association study using 658,385 single nucleotide polymorphisms (SNPs). The study population consisted of 3579 bulls of the FV breed with a total of 320,186 progeny with phenotypes for ACOP. The proportion of progeny with ACOP was used as a quantitative trait with high heritability (h^2^ = 0.79). A variance component based approach to account for population stratification uncovered twelve QTL regions on seven chromosomes. The identified QTL point to *MCM6*, *PAX3*, *ERBB3*, *KITLG*, *LEF1*, *DKK2*, *KIT*, *CRIM1*, *ATRN*, *GSDMC*, *MITF* and *NBEAL2* as underlying genes for eye area pigmentation in cattle. The twelve QTL regions explain 44.96% of the phenotypic variance of the proportion of daughters with ACOP. The chromosomes harbouring significantly associated SNPs account for 54.13% of the phenotypic variance, while another 19.51% of the phenotypic variance is attributable to chromosomes without identified QTL. Thus, the missing heritability amounts to 7% only. Our results support a polygenic inheritance pattern of ACOP in cattle and provide the basis for efficient genomic selection of animals that are less susceptible to serious eye diseases.

## Introduction

High-density SNP panels offer a new approach to deciphering the genetic architecture of complex traits [1] [2]. Large-scale genome-wide association studies (GWAS) have identified hundreds of variants contributing to the genetic variation of quantitative traits in humans, *e.g.* [3] [4]. Comprehensive GWAS have also been applied successfully to identify quantitative trait loci (QTL) for important traits in livestock species, frequently facilitated by using breeding values (or daughter yield deviations) *e.g.* [5], [2], [6]. Breeding values are highly heritable phenotypes as they are estimated on the basis of a large number of progeny records. Utilizing breeding values as phenotypes not only compensates smaller sample size in livestock GWAS [7] but also enables QTL mapping for traits which are recorded in the progeny.

Phenotypes for skin and coat pigmentation are readily accessible and accurately recordable traits with medium to high heritabilities [8] [9]. Skin and coat pigmentation traits have been studied and characterized extensively in humans (see [10] for a review), in laboratory animals [11] and in domestic animals (see [12] for a review). Variations of skin and coat colours naturally arose in the course of adaptation to altering environmental conditions, *e.g.* reaction to thermal stress [13] and increasing exposure to ultraviolet (UV) radiation [14].

Excessive exposure to UV radiation and a lack of ambilateral circumocular pigmentation (ACOP) are two predisposing factors to bovine ocular squamous cell carcinoma (BOSCC, eye cancer) [15] [16] [17]. Eye cancer is the most prevalent malignant tumour affecting cattle and causes substantial economic losses [16]. Breeds with white heads, *e.g.* Fleckvieh and Hereford, are particularly susceptible to BOSCC [18]. While the incidence of BOSCC in pertinently exposed Simmental (*i.e.* Fleckvieh) cattle is up to 53% [19], investigations concerning the prevalence of BOSCC in German herds have not been performed. Although there is evidence for a genetic predisposition to BOSCC, the heritability for BOSCC is low [20]. The susceptibility to BOSCC is considerably reduced in animals with ACOP, a highly heritable trait which can be easily identified [21]. Since the heritability for eye-area pigmentation is higher than for the susceptibility to BOSCC [20] [21], selection for ACOP is expected to rapidly decrease the number of affected animals [22] [17]. Furthermore, eye irritation and subsequent infection with bovine infectious keratoconjunctivitis (BIK, pinkeye) is more frequent in cattle without ACOP [23]. Selection for ACOP reduces the incidence of BIK and thus enhances animal welfare in areas with increased solar radiation. However, as excessive exposure to UV radiation is not a major challenge for German cattle, selection for ACOP does not take place in the German FV population.

The aim of the present study was to gain insights into the genetic architecture of a special aspect of skin pigmentation and to provide the basis for more efficient selection for animals that are less susceptible to serious eye diseases. Recording the pigmentation status in large progeny groups of artificial insemination bulls provided a highly heritable phenotype for a genome-wide association study. Using densely spaced SNPs, the association study identified twelve QTL regions.

## Results

**Figure 1:**
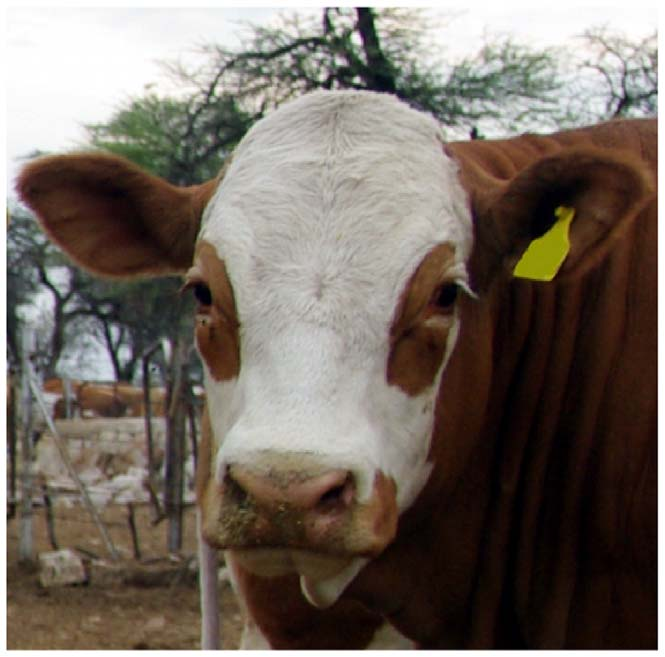
An animal of the dual-purpose Fleckvieh breed with ambilateral circumocular pigmentation. A white head is characteristic for animals of the Fleckvieh (FV) breed. However, in some half-sib families animals with pigmented skin around the eyes prevail. The pigmentation is restricted to the circumocular area and is not connected to the body pigmentation. Although there is variation regarding both the dimension and shape of ambilateral circumocular pigmentation (ACOP) in FV cattle, ACOP is routinely assessed as categorical trait only. The figure was kindly supplied by BAYERN-GENETIK GmbH (http://www.fleckvieh.de).

The proportion of daughters with ambilateral circumocular pigmentation (ACOP) (**Figure 1**) was obtained by phenotyping a median number of 59 daughters for 3579 bulls. It ranged from 0 to 69.1% with an average of 22.6% of the daughters per genotyped sire. After square root transformation, 66.67%, 95.87% and 99.89% of the values were within one, two and three standard deviations, respectively (**Figure S1)**. The resulting trait is a highly heritable progeny-derived phenotype for the bulls. Using the numerator relationship matrix among the 3579 animals built based on in-depth pedigree information in a random effect model, the heritability was estimated to be 0.79 (±0.04).

### Association study

**Figure 2:**
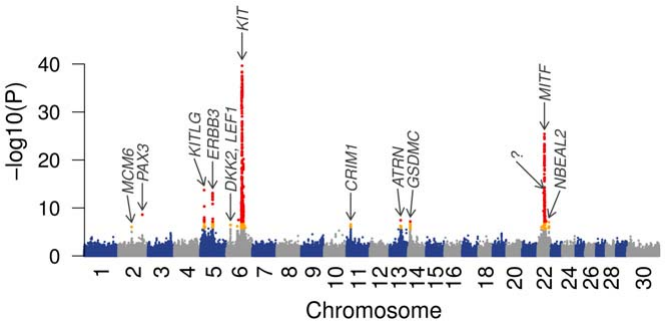
Manhattan plot of association of 658,385 SNPs with the proportion of daughters with ambilateral circumocular pigmentation in 3579 bulls of the Fleckvieh breed. The chromosomes are separated with alternating colours. Orange and red dots indicate chromosome-wide and genome-wide (P<7.59×10^−8^) significantly associated SNPs, respectively. The vertical axis is truncated for P values below -log10(10–41). Twelve identified QTL regions are indicated with arrows and gene identifiers.

The genome-wide association study based on a variance component based approach to account for population stratification identified twelve QTL regions on seven chromosomes (**Figure 2**). Among them, eight met the Bonferroni-corrected threshold for genome-wide significance, four were significantly associated on a chromosome-wide scale. A detailed overview of the characteristics of the identified QTL regions is given in **Table 1** and **Figure S2, S3, S4, S6, S7, S8, S9, S10, S11, S12, S13**. An additional analysis conditional on the most significantly associated SNP indicates the presence of a second independent QTL on BTA6 and BTA22, respectively (**Table 2** **, Figure S14 & S15**).

**Table 1:** The most significantly associated SNP for each of the twelve identified QTL regions for ambilateral circumocular pigmentation in the Fleckvieh breed.

Chromosome	SNP-id	Physical Position (bp)	Minor allele frequency	P	Candidate gene
2	BovineHD0200017704	61,628,137	0.38	9.01×10^−7^	*MCM6*
	UA-IFASA-5029	111,206,088	0.01	2.49×10^−9^	*PAX3*
5	BovineHD0500005310	18,206,817	0.13	1.90×10^−14^	*KITLG*
	BovineHD0500016261	57,554,914	0.32	7.59×10^−14^	*ERBB3*
6	BovineHD0600005244	18,975,451	0.43	3.51×10^−7^	*DKK2, LEF1*
	BTB-00263209	72,382,208	0.13	2.46×10^−73^	*KIT*
11	BTB-00753516	19,344,832	0.32	2.72×10^−7^	*CRIM1*
13	BovineHD1300014790	51,984,994	0.23	3.72×10^−8^	*ATRN*
14	Hapmap22917-BTC-068800	12,075,830	0.33	6.75×10^−8^	*GSDMC*
22	BovineHD2200008080	27,931,961	0.02	7.32×10^−14^	*-*
	BovineHD2200009208	32,245,023	0.17	3.75×10^−26^	*MITF*
	BovineHD2200015054	53,016,253	0.2	9.18×10^−8^	*NBEAL2*

The SNPs are arranged according to their physical position based on the UMD3.1 assembly of the bovine genome. The P values were obtained by using a variance components based approach to account for population stratification.

**Table 2:** The most significantly associated SNPs on chromosomes 6 and 22 after analysis conditional on the top SNP.

Chromosome	SNP-id	Physical Position (bp)	Minor allele frequency	P	Candidate gene
6	BovineHD0600020013	72,025,871	0.07	1.11×10^−11^	*KIT*
22	BovineHD4100015611	32,787,124	0.03	6.11×10^−17^	*MITF*

The SNPs are arranged according to their physical position based on the UMD3.1 assembly of the bovine genome. The P values are obtained by using a variance components based approach to account for population stratification conditional on the most significantly associated SNP for BTA6 (BTB-00263209) and BTA22 (BovineHD2200009208), respectively.

### Identification of functional genes within the associated regions

The gene content of the associated regions was analysed based on the University of Maryland UMD3.1 assembly of the bovine genome [24]. Strong association was observed in close vicinity to *KIT* (BTA6), *KITLG, ERBB3* (BTA5) and *MITF* (BTA22), four genes which play central roles in the migration of melanoblast cells and melanocyte development [25] [26] [27]. Two QTL on BTA2 point to *PAX3* and *MCM6* as candidate genes for ACOP in cattle. *PAX3* is a transcription factor known to be involved in melanogenesis [28]. *MCM6* is up regulated during pheomelanogenesis [29]. A QTL on BTA6, although only associated on a chromosome-wide level, is located between *DKK2* and *LEF1*. *DKK2* plays an essential role during eye development [30]. *LEF1* interacts with *MITF* via Wnt signalling [31]. The QTL on BTA11 is in close vicinity to *CRIM1*, which is up regulated in developing ocular tissues [32]. *ATRN*, a candidate gene for the BTA13 QTL, was shown to influence coat colour in mice [33]. On BTA14, association of a region containing *GSDMC* (alias *MLZE*) was observed. *MLZE* is up regulated in growing metastatic melanomas and is supposed to be important for melanoma progression [34]. A QTL on BTA22 identifies *NBEAL2* as candidate gene for ACOP. *NBEAL2* shows homology to *LYST*, which is responsible for pigmentation defects in humans and mice [35] [36]. We found no gene in immediate vicinity of the third BTA22 QTL.

### Allelic effects of significantly associated SNPs

**Figure 3:**
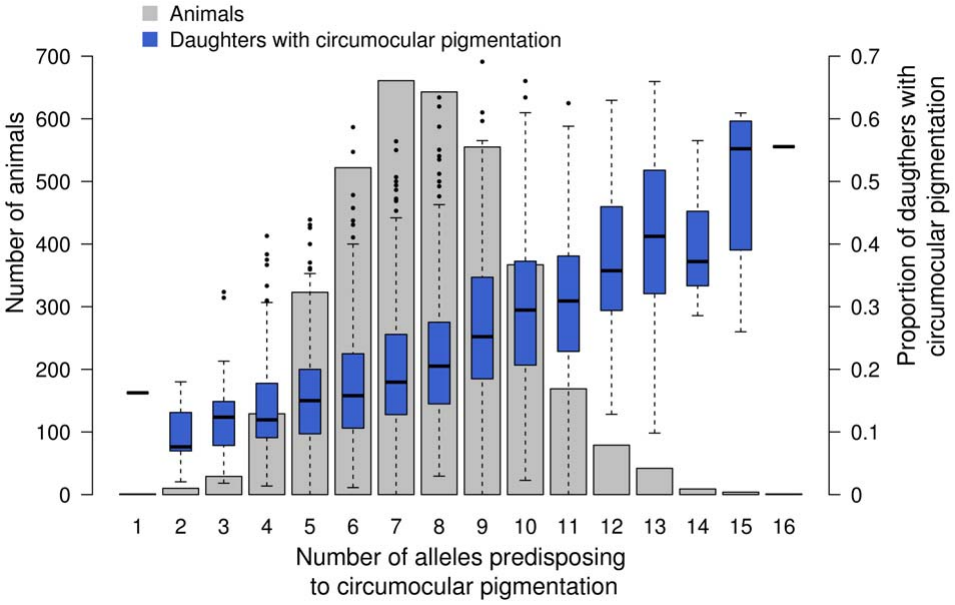
The effect of 14 significantly associated SNPs on the proportion of daughters with ambilateral circumocular pigmentation. 3579 Fleckvieh animals are grouped according to the number of alleles that predispose to ambilateral circumocular pigmentation (ACOP). The blue boxplots represent the proportion of daughters with ACOP for each group. The grey bars indicate the number of sires with an increasing number of predisposing alleles.

Alleles raising the proportion of daughters with ACOP were determined for the most significantly associated SNP for each QTL (**Table 1** **&** **Table 2**). **Figure 3** shows the frequency distribution of animals with an increasing number of alleles (from 1 to 16) predisposing to an increased number of progeny with ACOP. The proportion increases nearly linearly with an increasing number of predisposing alleles. Bulls with at least 15 predisposing alleles had >50% progeny with ACOP while the fraction is <20% for sires with less than seven predisposing alleles.

### Variance explained by all SNPs

The genomic relationship matrix, which was also applied for the genome-wide association study, was fitted in a mixed linear model to estimate the proportion of phenotypic variation accounted for by all 658,385 SNPs. To quantify the benefit of a high-density SNP map, the genomic relationship based on only 43,029 SNPs, which were genotyped in all animals, was estimated additionally. The very dense SNP map explains 73.64% of the phenotypic variation (*i.e.* 93.22% of the heritability) while the medium-dense map explains 69.97% (*i.e.* 88.56% of the heritability) (**Figure S16**).

### Partitioning of the genetic variation

**Figure 4:**
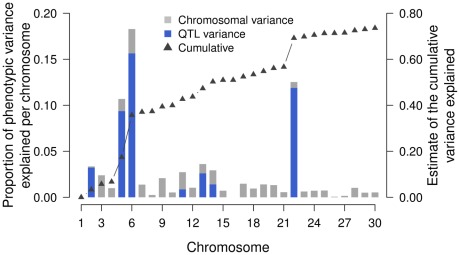
Chromosomal partitioning of the phenotypic variance. The grey and blue bars indicate the fraction of phenotypic variance attributed to a particular chromosome and QTL region, respectively. The triangles represent the cumulative proportion of phenotypic variance attributable to the 30 chromosomes.

We next built genomic relationship matrices among the 3579 animals for each chromosome separately in order to partitioning the phenotypic variation onto different chromosomes. The proportion of phenotypic variation attributable to a particular chromosome was then estimated with the effects of all chromosomes fitted simultaneously. The contribution of particular chromosomes varies strongly (**Figure 4**). A major fraction of the phenotypic variation is attributable to BTA5 (10.68%), BTA6 (18.29%) and BTA22 (12.53%), three chromosomes harbouring at least two identified QTL for ACOP. Totally, the seven chromosomes with identified QTL account for 54.13% of the phenotypic variation. This fraction decreases to 50.82% when the genomic relationship matrices built based on SNPs of the medium-density datasets were fitted (**Figure S16**). There was no significant association between chromosome length (in Mb units) and the proportion of phenotypic variance explained (P = 0.22, r^2^ = 0.06) (**Figure S17**).

Twelve identified QTL regions totally account for 44.96% of the phenotypic variance (*i.e.* 56.91% of the heritability). A major fraction of the phenotypic variance is attributable to the QTL regions encompassing *KITLG* (7.87%), *KIT* (14.56%) and *MITF* (11.33%) on chromosomes 5, 6 and 22, respectively (**Figure 4** **,** **Table 3**). While the identified QTL on BTA2 and BTA22 almost account for the entire chromosome variance, the QTL on BTA11 and BTA14 explain only a minor part of the particular chromosome variance. A QTL on BTA22 explains only a marginal fraction of the phenotypic variation.

**Table 3:** Proportion of phenotypic variance attributable to the twelve identified QTL regions.

Chromosome	Candidate gene	QTL-region [Basepairs]	Number of SNPs within the QTL-region	Proportion of phenotypic variance explained [%]
2	*MCM6*	59,128,137–64,128,137	1541	1.092
	*PAX3*	108,706,088–113,706,088	1356	2.129
5	*KITLG*	15,706,817–20,706,817	1334	7.869
	*ERBB3*	55,054,914–60,054,914	820	1.493
6	*DKK2, LEF1*	16,475,451–21,475,451	1501	1.077
	*KIT*	69,882,208–74,882,208	1593	14.560
11	*CRIM1*	16,844,832–21,844,832	1514	0.857
13	*ATRN*	49,394,994–54,394,994	910	2.598
14	*GSDMC*	9,575,830–14,575,830	1384	1.398
22	*-*	25,431,961–30,431,961	1334	0.002
	*MITF*	29,745,023–34,745,023	1356	11.332
	*NBEAL2*	50,516,253–55,516,253	1507	0.548

A 5 Mb interval centred on the most significantly associated SNP was considered as QTL-region.

The genomic relationship matrix for each QTL was built based upon SNPs within the 5 Mb interval.

The proportion of phenotypic variance explained was then estimated with the effects off all chromosomes and QTL fitted simultaneously.

## Discussion

Coat colour phenotypes are routinely recorded for a large number of females, while genotyping is routinely performed in males in the FV breed. To benefit from the large number of cows with phenotypes and the substantial number of bulls with genotypes, the proportion of daughters with ACOP was assessed for a total of 320,186 cows sired by 3579 genotyped artificial insemination bulls. The resulting phenotypes can be considered as breeding values for the bulls. Using pedigree information, the heritability (*i.e.* reliability of the breeding value) for the proportion of daughters with ACOP was estimated as 0.79, which is considerably higher than previous estimates of the heritability for eye-area pigmentation in cattle [37] [17] [38]. A highly heritable phenotype facilitates QTL mapping considerably, especially if the number of genotyped individuals is limited [39]. Nine of the twelve identified QTL for ACOP account for at least 1% of the trait variance. Although the limited number of genotyped animals restricted the power to detect QTL with small effects, the present study identified three QTL that account for less than 1% of the trait variance.

The coat pigmentation phenotype assessed in this study is a quantitative trait with numerous loci with small effects and few loci with large effects. The twelve identified QTL regions can be considered as the major determinants for ACOP in cattle as they explain 44.96% of the phenotypic variation. Totally, the seven chromosomes with identified QTL account for 54.13% of the phenotypic variation. However, a substantial fraction of the phenotypic variation is attributable to chromosomes without identified QTL. Increasing the number of genotyped animals might enable the detection of additional QTL with minor effect sizes [40], however the number of detectable QTL is limited even in studies with very large sample sizes [41]. The present study nevertheless demonstrates both the leverage potential of progeny phenotyping and the utility of a dense marker map for unravelling the genetic architecture of complex traits in livestock animals. Totally, the 658,385 and 43,029 SNPs accounted for ∼93% and ∼89% of the heritability. These fractions are distinctly higher than those reported for traits with similar heritability in human genetics [42] [1]. Presumably due to the considerably lower number of independent chromosome segments in cattle, resulting from a small effective population size and concomitant substantial long-range linkage disequilibrium [39]. Our results display that applying dense SNP panels allows to capture most of the genetic variation of complex traits in highly structured livestock populations and thus reduces the *‘missing heritability’* [43].

High-density genotyping of a minor fraction of the animals of our study population enabled the accurate imputation of ∼96% of the genotypes for the remaining animals genotyped at a lower density (Figure S18). This agrees with findings in the American Holstein population [44]. The actual imputation accuracy for the complete study population is supposed to be even higher, since only half of the animals with high-density genotypes were applied as reference population for the evaluation of imputation accuracy whereas genotypes for the remaining animals were set to missing. Increasing the number of reference individuals with high-density genotypes enables a better resolution of the haplotype structure of the population implicating higher imputation accuracy [45] [46]. Our results demonstrate that the availability of a dense SNP panel and concomitant genotype imputation enables the mapping of QTL for complex traits in livestock populations at a better resolution.

Eight of the twelve identified QTL point to genes, *i.e. MCM6, PAX3, KITLG*, *ERBB3, KIT*, *ATRN*, *MITF* and *NBEAL2*, affecting various coat colour phenotypes in cattle and other species *via* pigment cell genesis and/or pigment formation (*e.g.* [29] [27] [25] for a review). Interestingly, two of the identified QTL for ACOP in the FV breed are in close vicinity to genes affecting eye morphogenesis during embryonal development, *i.e. DKK2* [30] and *CRIM1* [32]. The QTL on BTA14 points to *GSDMC* (alias *MLZE*) as candidate gene for ACOP in cattle. There are no clues for a direct contribution of *MLZE* to mammalian pigmentation traits or embryonal eye development. However, expression of *MLZE* in growing metastatic melanomas implies a contribution of *MLZE* to melanoma progression [34] [47] and thus possibly to normal melanocyte development.

The identified candidate genes for ACOP interact in a complex fashion, *e.g.* during melanocyte development and melanocyte migration [48] [49]. Two candidate genes for ACOP (*LEF1*, *PAX3*) encode transcription factors regulating the promoter for the BTA22 candidate gene *MITF* [49]. The BTA6 candidate gene *KIT* encodes a transmembrane receptor for the mast cell growth factor encoded by the BTA5 candidate gene *KITLG* [50]. Previous studies evidenced that *KIT* alleles acting in a dominant fashion completely inhibit pigmentation in pigs [51]. A similar mechanism is plausible for eye-area pigmentation in cattle. However, the present study accounts for additive effects only as the applied phenotype is recorded in the progeny of genotyped artificial insemination bulls. Direct phenotypes for ACOP were not available for the genotyped animals. The proportion of daughters with ACOP is a progeny-derived phenotype for the bulls and acts therefore purely additively. Thus, we cannot dissect non-additive effects, although they are likely to explain a substantial fraction of the genetic variation for complex traits, such as coat colour [52] [53]. Assessing phenotypes for ACOP in the genotyped male animals might enable to quantify the extent of non-additive effects. However, the identification of non-additive effects on a genome-wide scale requires large sample sizes and is computationally demanding [54] [55]. Investigating causal variants directly for non-additive effects overcomes the substantial burden of multiple testing and concomitantly restricts computational costs [56]. However, the present study illustrates the complexity in revealing causal variants in livestock populations. Significantly associated QTL regions might expand over several million base pairs due to extensive linkage disequilibrium (*e.g.* Figure S7), rendering the identification of underlying variants/mechanisms a difficult task. Access to large independent validation populations [57] and comprehensive functional investigations [58], respectively, is indispensable for the fine-mapping of QTL regions in livestock populations.

Pigmentation around the eyes is highly correlated with eye-lid and corneoscleral pigmentation [22]. Corneoscleral pigmentation considerably reduces the susceptibility to bovine infectious keratoconjunctivitis (BIK) [23] and eye cancer (BOSCC) [59]. In the present study, the number of progeny with ACOP increased to >50% with an increasing number of favourable QTL alleles of the sire. The selection of bulls based on these QTL alleles might rapidly increase the number of progeny with ACOP and thus contribute to reducing the incidence of BIK and BOSCC in areas of increased solar radiation [21]. However, since the twelve identified QTL regions account for 56.9% of the heritability only, genome-wide evaluation of sires using the entire set of high-density SNPs should allow to most efficiently increase the proportion of progeny with ACOP and thus should reduce the incidence of serious health problems in cattle.

## Materials and Methods

### Animals and phenotypes

The proportion of daughters with ACOP (**Figure 1**) was assessed for 3579 progeny tested bulls of the Fleckvieh (FV) breed. Eye-area pigmentation is routinely recorded during the examination of first-crop daughters of test bulls as a categorical trait. However, phenotypes for ACOP are not recorded routinely for male animals. Phenotypic records for 320,186 FV cows were provided from the Bavarian State Research Center for Agriculture (http://www.lfl.bayern.de). The number of daughters per sire ranged from 20 to 3949 with a median of 59 daughters. An approximately normally distributed phenotype was obtained by square root transformation of the proportion of daughters with ACOP (**Figure S1**). Genetic parameters were estimated using the random effect model 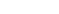 [60], where y is the square root transformed proportion of daughters with ACOP, g is a vector of random genetic effects and e is a vector of random residual deviates (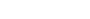). g is normally distributed (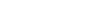), where 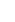 is the genetic variance and A is the numerator relationship matrix among the 3579 bulls tracing back pedigree information to 1920.

### Genotypes and quality control

Genotyping was performed with three different genotyping arrays. 3387 FV bulls were genotyped with the Illumina BovineSNP 50K Bead chip® interrogating 54,001 (version 1, 54K*v1*) and 54,609 (version 2, 54K*v2*) SNPs, respectively. Additionally, 810 FV bulls were genotyped with the Illumina BovineHD Bead chip® interrogating 777,962 SNPs (777K). 521 bulls of the 777K data set were also genotyped with the 54Kv1 genotyping array (**Table S1, S2, S3**). The chromosomal position of the SNPs was determined according to the University of Maryland UMD3.1 assembly of the bovine genome sequence [24]. Quality control was performed for the three datasets separately using *PLINK* [61]. Animals with more than 10% missing genotypes were not considered for further analyses. Those SNPs with unknown, Y-chromosomal or mitochondrial position or if genotyping failed in more than 10% of the animals were excluded. Additionally, SNPs with a minor allele frequency (MAF) <0.5% and SNPs showing significant (P<0.0001) deviation from the Hardy-Weinberg Equilibrium were omitted for subsequent analysis. The genomic relationship was calculated as proposed by VanRaden [62] and compared with the pedigree relationship. Animals showing major discrepancies of the pedigree and genomic relationship were omitted. A detailed overview of the number of SNPs and animals not passing the quality criteria is given in **Table S1**.

### *In silico* genotyping

A subset of 38,820 SNPs was interrogated with all three genotyping arrays. However, genotypes for 488, 394 and 611,702 SNPs were exclusively interrogated with the 54K*v1*, 54K*v2* and 777K arrays, respectively (**Table S2 & S3**). The datasets were combined and missing genotypes were inferred using *findhapV2* [44]. After genotype imputation, the resulting dataset comprised 3643 animals and 658,385 SNPs with an average genotyping rate of 99.68% per individual. Progeny records were available for 3579 individuals only.

### Evaluation of imputation accuracy

Imputation accuracy was evaluated within 802 animals of the high-density dataset to assess the quality of the imputed genotypes. We randomly selected 400 animals as reference population with full genotype information. Genotypes were masked for the remaining 402 animals for all SNPs except for 40,062 SNPs interrogated by the 54K*v1* Bead Chip. Genotypes for the 613,232 masked SNPs were subsequently inferred using *findhapV2* and compared with the true genotypes. The number of SNPs used for the evaluation of the imputation accuracy is given for each chromosome in **Table S4**. In total, 99.52% of the genotypes could be inferred with an average genotypic concordance of 95.78%. The allele frequency was the major determinant for imputation accuracy (**Figure S18**).

### Genome-wide association study

A genome-wide association study was performed using a variance components based approach to account for population stratification and to eliminate the resulting inflation of false positive associations. We used *EMMAX* [63] to fit the model 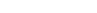, where Y is the square root transformed proportion of daughters with ACOP, b is the SNP effect, X is a design matrix of SNP genotypes, u is the additive genetic effect 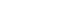, where 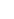 is the additive genetic variance and G is the genomic relationship matrix (GRM) among the 3579 animals with phenotype information built based on 658,385 SNPs following VanRaden's approach (see above). SNPs were considered as significantly associated on a genome-wide level for P values below 7.59×10^−8^ (Bonferroni-corrected type I error threshold for 658,385 independent tests).

### Estimating the power of the genome-wide association study

The required sample size (N) for a GWAS to identify a QTL explaining a given fraction (q^2^) of the trait variance can be estimated as 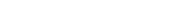, where 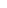 is the significance level, z is the normal score and 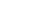 is the power to detect association [64]. Considering 3579 animals and the Bonferroni-corrected significance threshold for an average number of 22.000 tests per chromosome, the power to detect a QTL accounting for at least 1% of the trait variance is approximately 90% in the present study.

### Partitioning of the genetic variance

In order to estimate the proportion of phenotypic variance attributed to a particular chromosome, a GRM was built (see above) for each of the 30 chromosomes separately. We used *GCTA* [65] to fit the model 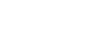, where y is a vector of the square root transformed proportion of daughters with ACOP, g is a vector of genetic effects attributed to the i^th^ chromosome, and e is a vector of random residual deviates. 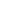 is assumed to be normally distributed with 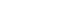, where 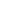 is the GRM built based on SNPs on the i^th^ chromosome. Variance components were estimated with the effects of all chromosomes fitted simultaneously and the proportion of phenotypic variance attributable to the i^th^ chromosome was calculated as 
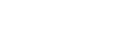
. To estimate the proportion of phenotypic variance explained by each of the twelve identified QTL regions, SNPs within a 5 Mb interval centred on the most significantly associated SNP were considered for building the GRM for each QTL region. All SNPs except those within the 5 Mb interval were used to build the GRM for the chromosome harbouring the QTL. Variance components were estimated with the effects of all 30 chromosomes and twelve QTL regions fitted simultaneously (see above).

## Supporting Information

Figure S1 **Distribution of the applied phenotype.** The boxplot (A) and histogram (B) display the distribution of the square root transformed proportion of daughters with ambilateral circumocular pigmentation. The deviation from the expected Gaussian normal distribution is only marginal (C). 66.67%, 95.87% and 99.89% of the values are within one two and three standard deviations, respectively.
(PDF)

Figure S2 **Detailed overview of the identified QTL region on BTA2 (61,300,000 bp–62,300,000 bp).** The gene content was assessed based on the University of Maryland (UMD3.1) assembly of the bovine genome. The putative functional candidate gene is highlighted with red colour. The diamond represents the most significantly associated SNP while different colours represent the linkage disequilibrium (r^2^) between the most significantly associated SNP and all other SNPs within the displayed region. The heatmap displays the pairwise linkage disequilibrium.
(PDF)

Figure S3 **Detailed overview of the identified QTL region on BTA2 (110,600,000 bp–111,600,000 bp).** The gene content was assessed based on the University of Maryland (UMD3.1) assembly of the bovine genome. The putative functional candidate gene is highlighted with red colour. The diamond represents the most significantly associated SNP while different colours represent the linkage disequilibrium (r^2^) between the most significantly associated SNP and all other SNPs within the displayed region. The heatmap displays the pairwise linkage disequilibrium.
(PDF)

Figure S4 **Detailed overview of the identified QTL region on BTA5 (17,800,000 bp–18,800,000 bp).** The gene content was assessed based on the University of Maryland (UMD3.1) assembly of the bovine genome. The putative functional candidate gene is highlighted with red colour. The diamond represents the most significantly associated SNP while different colours represent the linkage disequilibrium (r^2^) between the most significantly associated SNP and all other SNPs within the displayed region. The heatmap displays the pairwise linkage disequilibrium.
(PDF)

Figure S5 **Detailed overview of the identified QTL region on BTA5 (57,200,000 bp–57,800,000 bp).** The gene content was assessed based on the University of Maryland (UMD3.1) assembly of the bovine genome. The putative functional candidate gene is highlighted with red colour. The diamond represents the most significantly associated SNP while different colours represent the linkage disequilibrium (r^2^) between the most significantly associated SNP and all other SNPs within the displayed region. The heatmap displays the pairwise linkage disequilibrium.
(PDF)

Figure S6 **Detailed overview of the identified QTL region on BTA6 (18,000,000 bp–20,000,000 bp).** The gene content was assessed based on the University of Maryland (UMD3.1) assembly of the bovine genome. The putative functional candidate gene is highlighted with red colour. The diamond represents the most significantly associated SNP while different colours represent the linkage disequilibrium (r^2^) between the most significantly associated SNP and all other SNPs within the displayed region. The heatmap displays the pairwise linkage disequilibrium.
(PDF)

Figure S7 **Detailed overview of the identified QTL region on BTA6 (71,500,000 bp–73,000,000 bp).** The gene content was assessed based on the University of Maryland (UMD3.1) assembly of the bovine genome. The putative functional candidate gene is highlighted with red colour. The diamond represents the most significantly associated SNP while different colours represent the linkage disequilibrium (r^2^) between the most significantly associated SNP and all other SNPs within the displayed region. The heatmap displays the pairwise linkage disequilibrium.
(PDF)

Figure S8 **Detailed overview of the identified QTL region on BTA11 (18,000,000 bp–20,000,000 bp).** The gene content was assessed based on the University of Maryland (UMD3.1) assembly of the bovine genome. The putative functional candidate gene is highlighted with red colour. The diamond represents the most significantly associated SNP while different colours represent the linkage disequilibrium (r^2^) between the most significantly associated SNP and all other SNPs within the displayed region. The heatmap displays the pairwise linkage disequilibrium.
(PDF)

Figure S9 **Detailed overview of the identified QTL region on BTA13 (51,500,000 bp–52,500,000 bp).** The gene content was assessed based on the University of Maryland (UMD3.1) assembly of the bovine genome. The putative functional candidate gene is highlighted with red colour. The diamond represents the most significantly associated SNP while different colours represent the linkage disequilibrium (r^2^) between the most significantly associated SNP and all other SNPs within the displayed region. The heatmap displays the pairwise linkage disequilibrium.
(PDF)

Figure S10 **Detailed overview of the identified QTL region on BTA14 (11,500,000 bp–13,000,000 bp).** The gene content was assessed based on the University of Maryland (UMD3.1) assembly of the bovine genome. The putative functional candidate gene is highlighted with red colour. The diamond represents the most significantly associated SNP while different colours represent the linkage disequilibrium (r^2^) between the most significantly associated SNP and all other SNPs within the displayed region. The heatmap displays the pairwise linkage disequilibrium.
(PDF)

Figure S11 **Detailed overview of the identified QTL region on BTA22 (27,000,000 bp–29,000,000 bp).** The gene content was assessed based on the University of Maryland (UMD3.1) assembly of the bovine genome. The putative functional candidate gene is highlighted with red colour. The diamond represents the most significantly associated SNP while different colours represent the linkage disequilibrium (r^2^) between the most significantly associated SNP and all other SNPs within the displayed region. The heatmap displays the pairwise linkage disequilibrium.
(PDF)

Figure S12 **Detailed overview of the identified QTL region on BTA22 (31,000,000 bp–34,000,000 bp).** The gene content was assessed based on the University of Maryland (UMD3.1) assembly of the bovine genome. The putative functional candidate gene is highlighted with red colour. The diamond represents the most significantly associated SNP while different colours represent the linkage disequilibrium (r^2^) between the most significantly associated SNP and all other SNPs within the displayed region. The heatmap displays the pairwise linkage disequilibrium.
(PDF)

Figure S13 **Detailed overview of the identified QTL region on BTA22 (52,500,000 bp–53,500,000 bp).** The gene content was assessed based on the University of Maryland (UMD3.1) assembly of the bovine genome. The putative functional candidate gene is highlighted with red colour. The diamond represents the most significantly associated SNP while different colours represent the linkage disequilibrium (r^2^) between the most significantly associated SNP and all other SNPs within the displayed region. The heatmap displays the pairwise linkage disequilibrium.
(PDF)

Figure S14 **Association of 30,985 SNPs on Chromosome 6 with ambilateral circumocular pigmentation in 3579 animals of the Fleckvieh population.** Results for the initial analysis (A) and for the analysis conditional on the *BTB-00263209* SNP (B). Orange dots represent significantly associated SNPs on a chromosome-wide level, red dots represent significantly associated SNPs on a genome-wide level.
(PDF)

Figure S15 **Association of 16,722 SNPs on Chromosome 22 with ambilateral circumocular pigmentation in 3579 animals of the Fleckvieh population.** Results for the initial analysis (A) and for the analysis conditional on the *BovineHD2200009208* SNP (B). Orange dots represent significantly associated SNPs on a chromosome-wide scale, red dots represent significantly associated SNPs on a genome-wide scale.
(PDF)

Figure S16 **Effect of different marker densities on the chromosomal partitioning of the genetic variance.** The grey and blue bars indicate the fraction of phenotypic variance attributed to a particular chromosome using the genomic relationship matrices built based on medium-density and high-density SNP information, respectively.
(PDF)

Figure S17 **Correlation between chromosome length and the fraction of phenotypic variance explained.** The estimate of the proportion of phenotypic variance explained by a particular chromosome is displayed as a function of the physical chromosome length (in Mb units). Red numbers indicate chromosomes with identified QTL. The blue line is a linear regression line with slope 3.2×10^−4^ (r^2^ = 0.06).
(PDF)

Figure S18 **Accuracy of the imputed genotypes.** Imputation accuracy was assessed based on genotypes of 613,232 chromosome-wide distributed SNPs of 402 animals. The proportion of correctly imputed alleles is displayed as a function of the allele frequency. The boxplots show the results for allele frequency bins of 2.5%. The concordance between imputed and true allele was poor (41.06%) for rare alleles (*i.e.* alleles with a frequency <2.5%), while imputation of frequent alleles (*i.e.* alleles with a frequency >65%) resulted in an allelic concordance >99%.
(PDF)

Table S1 **Number of SNPs not passing the quality control parameters for the medium-density (54K** ***v1*** **, 54K** ***v1*** **) and the high-density (777K) datasets.** The number of SNPs and animals not passing the applied quality parameters as well as the final number of SNPs and animals is given for the two medium-density (54K*v1*, 54K*v2*) and for the high-density (777K) dataset, respectively (some SNPs failed for more than one quality control parameter).
(PDF)

Table S2 **Number of SNPs for each of the three datasets after quality control.** Numbers along the diagonal represent the final number of SNPs for the two medium-density (54K*v1*, 54K*v2*) and for the high-density (777K) dataset, respectively. Off-diagonal numbers indicate the intersection.
(PDF)

Table S3 **Number of animals for each of the three datasets after quality control.** Numbers along the diagonal represent the final number of SNPs for the two medium-density (54K*v1*, 54K*v2*) and for the high-density (777K) dataset, respectively. Off-diagonal numbers indicate the intersection.
(PDF)

Table S4 **Number of SNPs used for the evaluation of the imputation accuracy.**
(PDF)
