# Supplementary figures and images for: Identification of QTL for UV-Protective Eye Area Pigmentation in Cattle by Progeny Phenotyping and Genome-Wide Association Analysis
Source: PLoS One. 2012 May 2;7(5):e36346. doi: 10.1371/journal.pone.0036346 (PMC3342244; doi:10.1371/journal.pone.0036346)

A

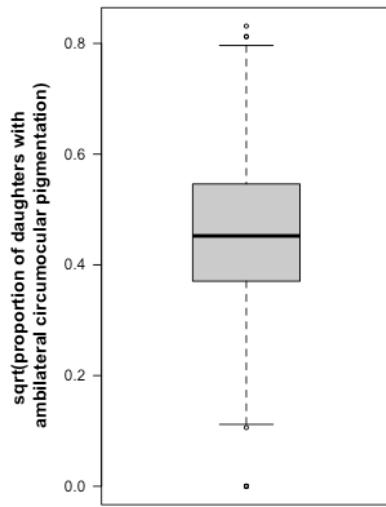

B

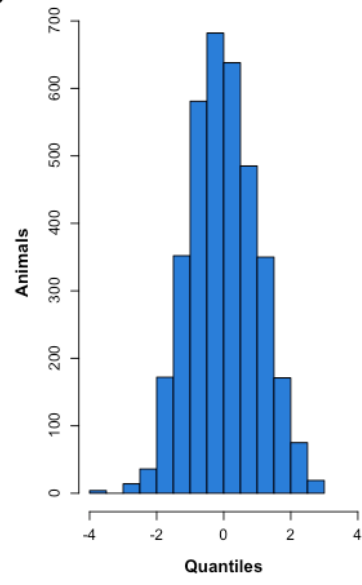

C

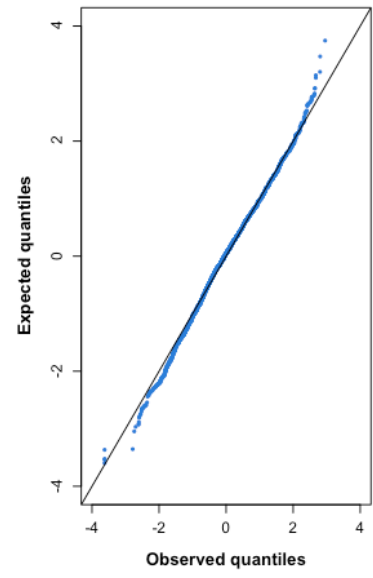

Supplement: Figure S1 — Distribution of the applied phenotype. The boxplot (A) and histogram (B) display the distribution of the square root transformed proportion of daughters with ambilateral circumocular pigmentation. The deviation from the expected Gaussian normal distribution is only marginal (C). 66.67%, 95.87% and 99.89% of the values are within one two and three standard deviations, respectively. (PDF) [file pone.0036346.s001.pdf]

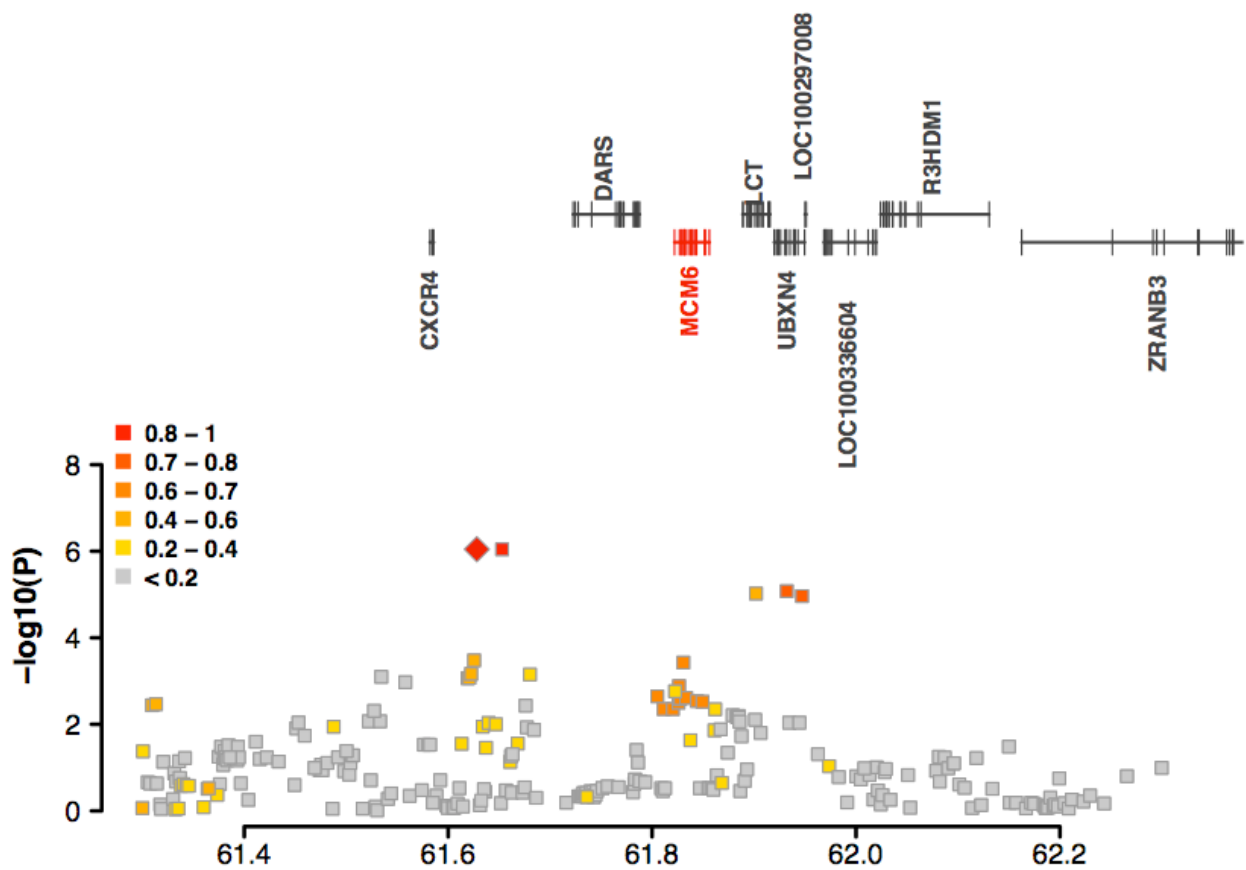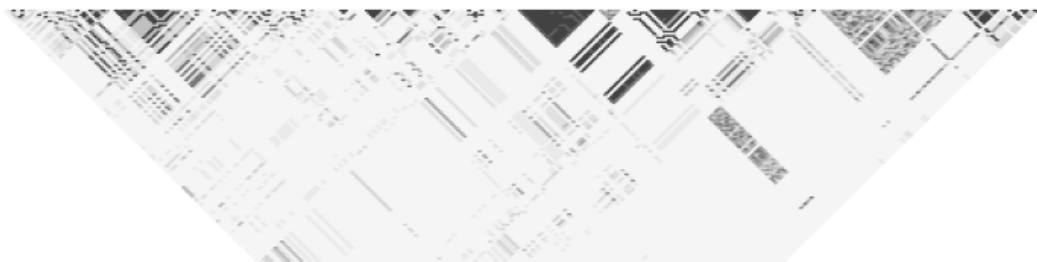

Supplement: Figure S2 — Detailed overview of the identified QTL region on BTA2 (61,300,000 bp–62,300,000 bp). The gene content was assessed based on the University of Maryland (UMD3.1) assembly of the bovine genome. The putative functional candidate gene is highlighted with red colour. The diamond represents the most significantly associated SNP while different colours represent the linkage disequilibrium (r2) between the most significantly associated SNP and all other SNPs within the displayed region. The heatmap displays the pairwise linkage disequilibrium. (PDF) [file pone.0036346.s002.pdf]

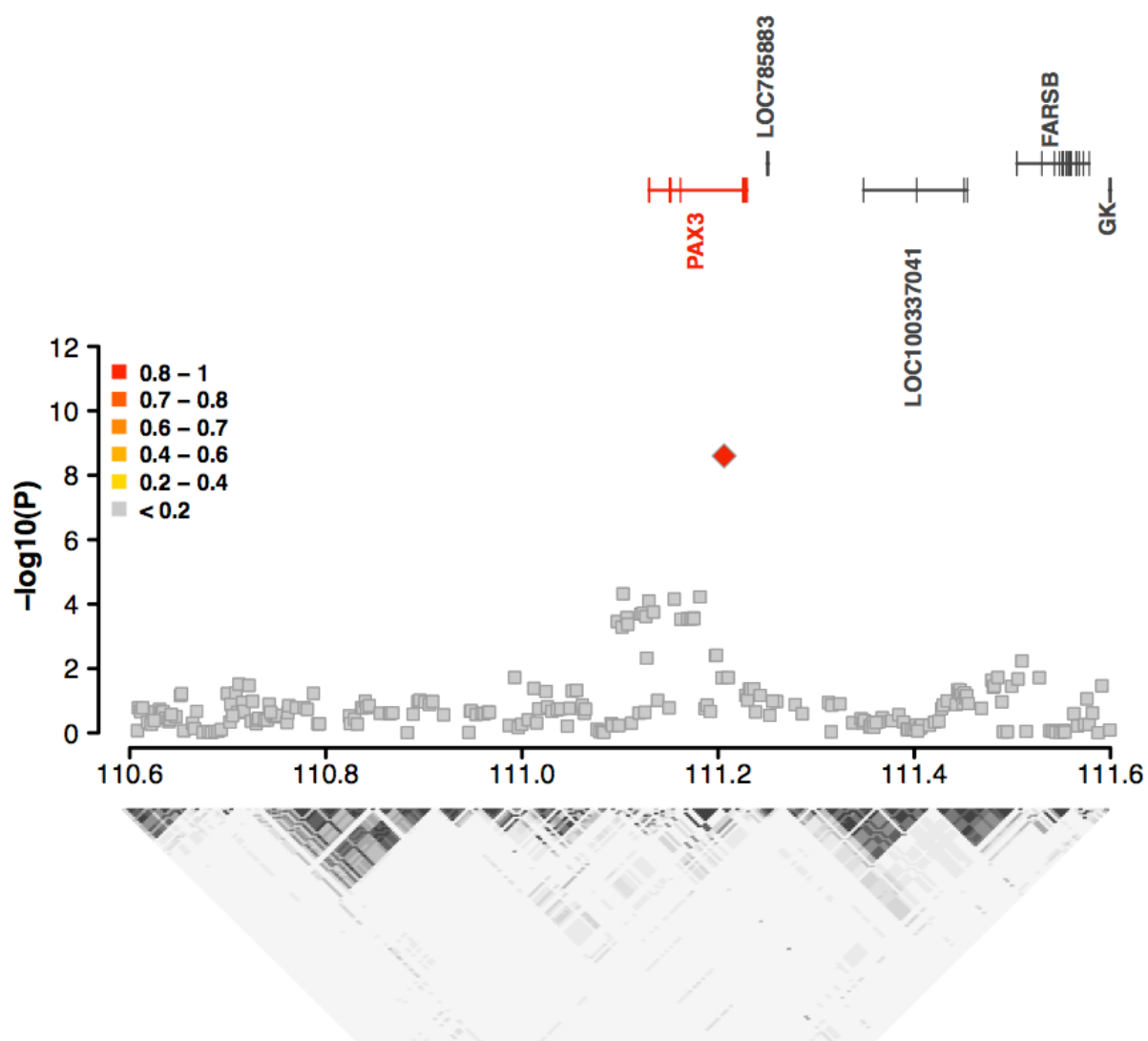

Supplement: Figure S3 — Detailed overview of the identified QTL region on BTA2 (110,600,000 bp–111,600,000 bp). The gene content was assessed based on the University of Maryland (UMD3.1) assembly of the bovine genome. The putative functional candidate gene is highlighted with red colour. The diamond represents the most significantly associated SNP while different colours represent the linkage disequilibrium (r2) between the most significantly associated SNP and all other SNPs within the displayed region. The heatmap displays the pairwise linkage disequilibrium. (PDF) [file pone.0036346.s003.pdf]

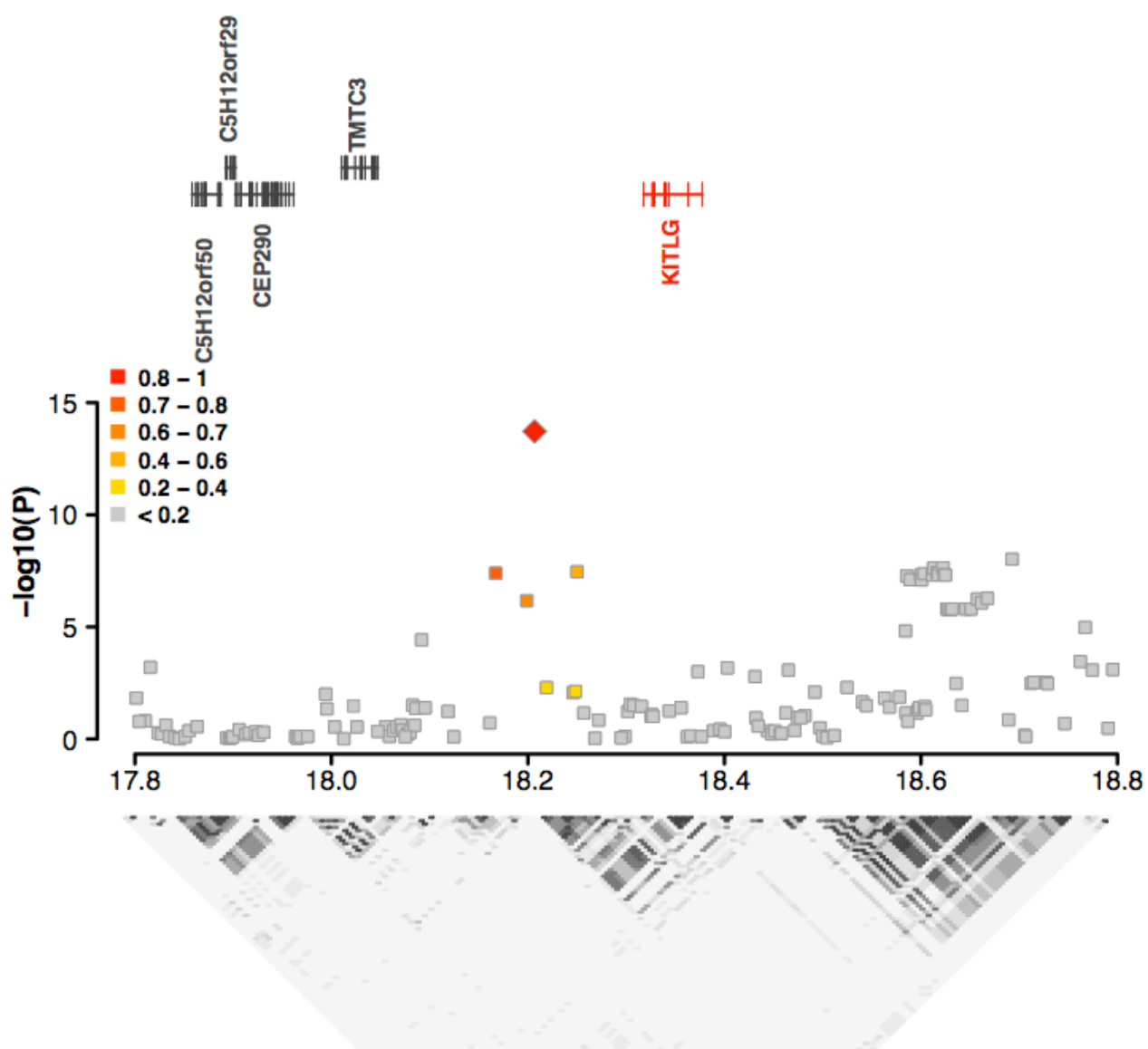

Supplement: Figure S4 — Detailed overview of the identified QTL region on BTA5 (17,800,000 bp–18,800,000 bp). The gene content was assessed based on the University of Maryland (UMD3.1) assembly of the bovine genome. The putative functional candidate gene is highlighted with red colour. The diamond represents the most significantly associated SNP while different colours represent the linkage disequilibrium (r2) between the most significantly associated SNP and all other SNPs within the displayed region. The heatmap displays the pairwise linkage disequilibrium. (PDF) [file pone.0036346.s004.pdf]

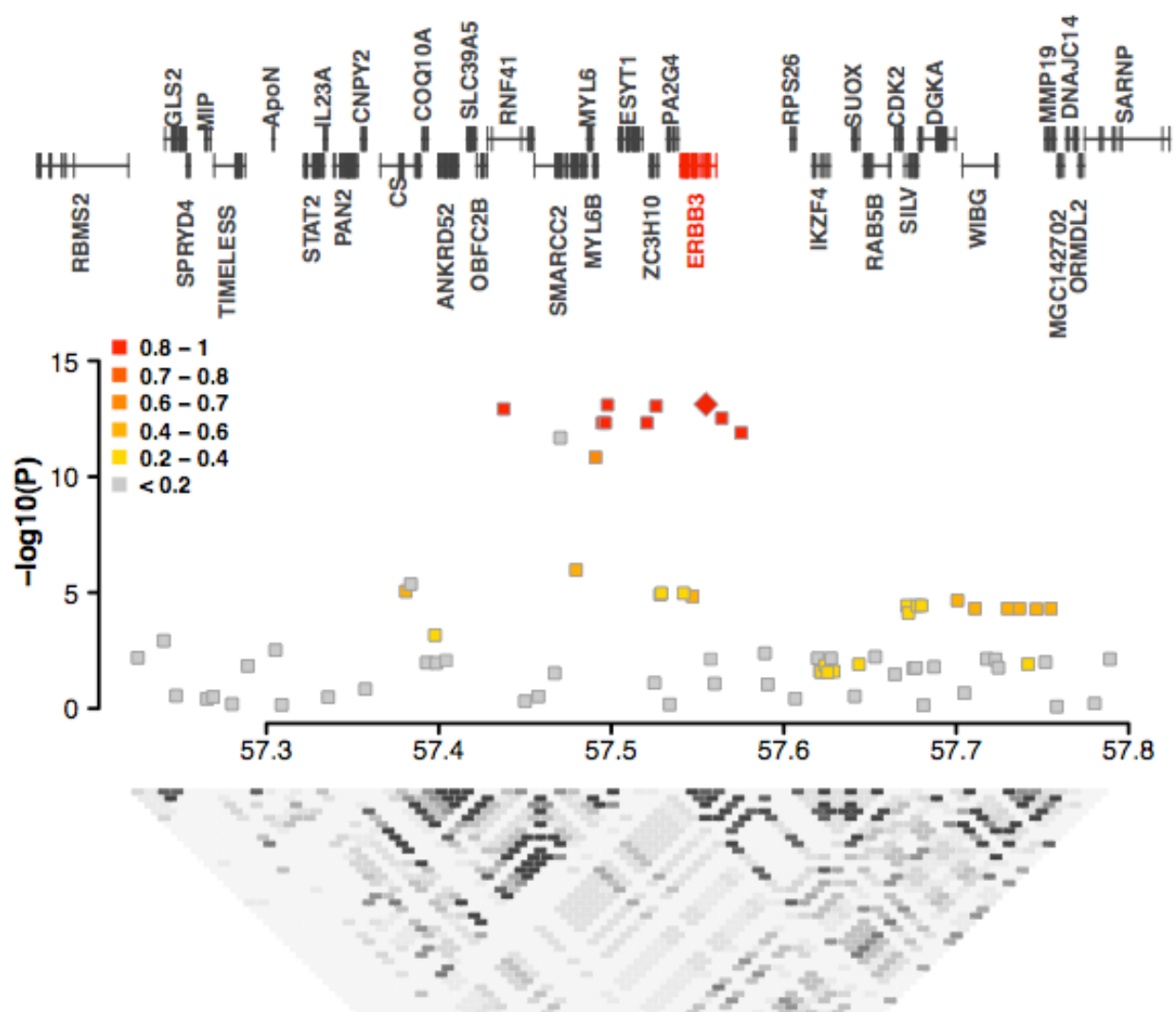

Supplement: Figure S5 — Detailed overview of the identified QTL region on BTA5 (57,200,000 bp–57,800,000 bp). The gene content was assessed based on the University of Maryland (UMD3.1) assembly of the bovine genome. The putative functional candidate gene is highlighted with red colour. The diamond represents the most significantly associated SNP while different colours represent the linkage disequilibrium (r2) between the most significantly associated SNP and all other SNPs within the displayed region. The heatmap displays the pairwise linkage disequilibrium. (PDF) [file pone.0036346.s005.pdf]

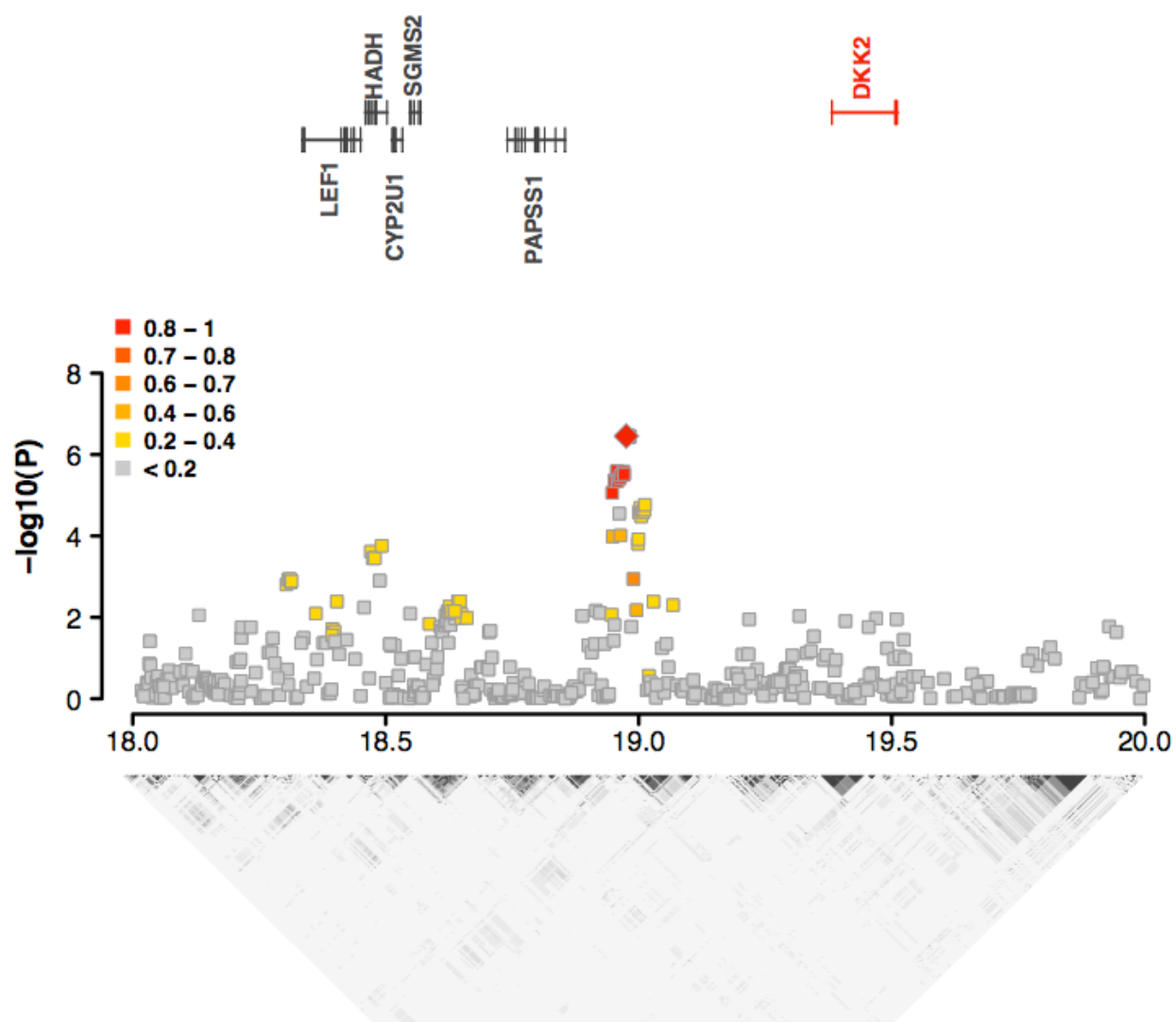

Supplement: Figure S6 — Detailed overview of the identified QTL region on BTA6 (18,000,000 bp–20,000,000 bp). The gene content was assessed based on the University of Maryland (UMD3.1) assembly of the bovine genome. The putative functional candidate gene is highlighted with red colour. The diamond represents the most significantly associated SNP while different colours represent the linkage disequilibrium (r2) between the most significantly associated SNP and all other SNPs within the displayed region. The heatmap displays the pairwise linkage disequilibrium. (PDF) [file pone.0036346.s006.pdf]

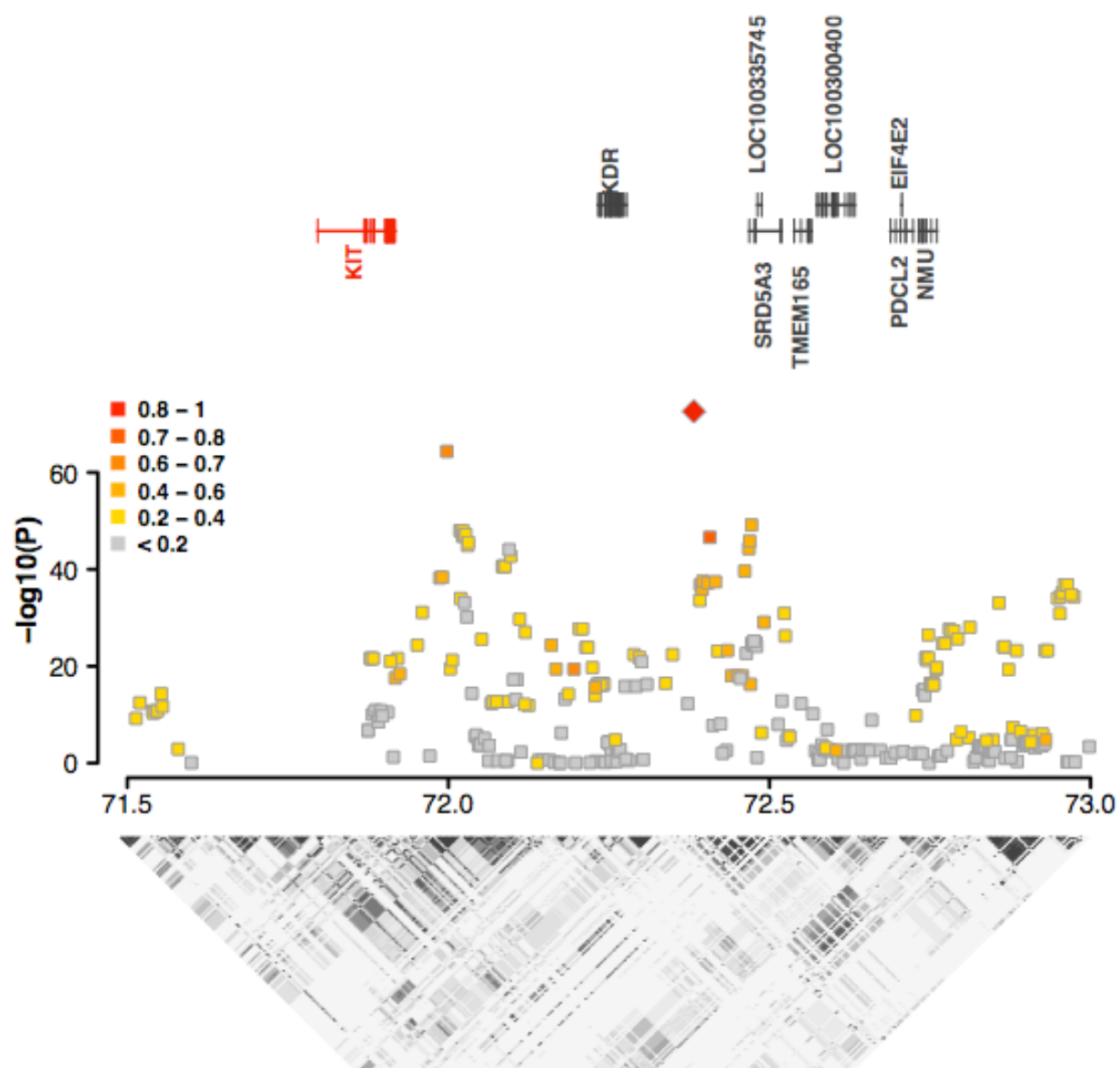

Supplement: Figure S7 — Detailed overview of the identified QTL region on BTA6 (71,500,000 bp–73,000,000 bp). The gene content was assessed based on the University of Maryland (UMD3.1) assembly of the bovine genome. The putative functional candidate gene is highlighted with red colour. The diamond represents the most significantly associated SNP while different colours represent the linkage disequilibrium (r2) between the most significantly associated SNP and all other SNPs within the displayed region. The heatmap displays the pairwise linkage disequilibrium. (PDF) [file pone.0036346.s007.pdf]

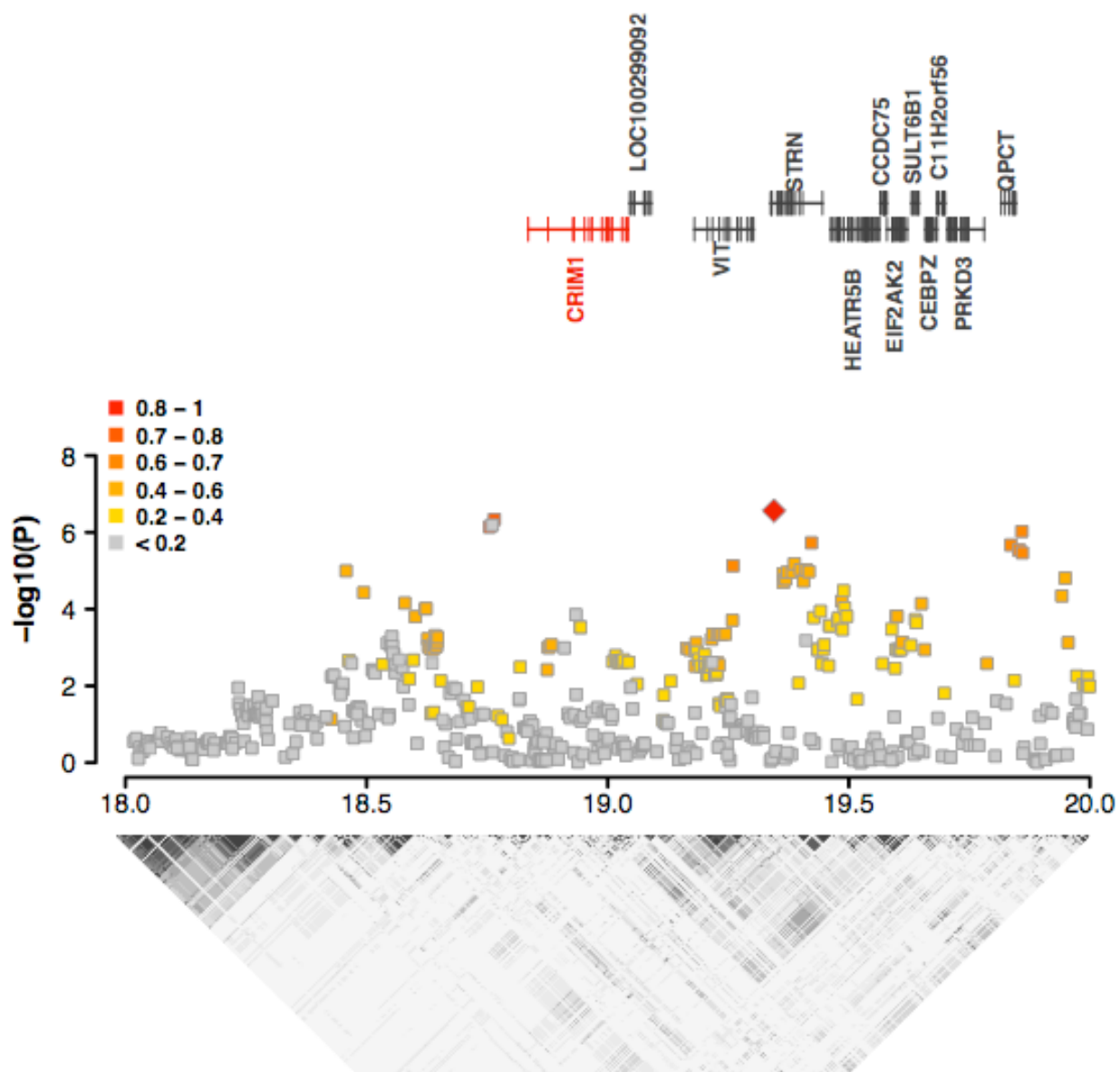

Supplement: Figure S8 — Detailed overview of the identified QTL region on BTA11 (18,000,000 bp–20,000,000 bp). The gene content was assessed based on the University of Maryland (UMD3.1) assembly of the bovine genome. The putative functional candidate gene is highlighted with red colour. The diamond represents the most significantly associated SNP while different colours represent the linkage disequilibrium (r2) between the most significantly associated SNP and all other SNPs within the displayed region. The heatmap displays the pairwise linkage disequilibrium. (PDF) [file pone.0036346.s008.pdf]

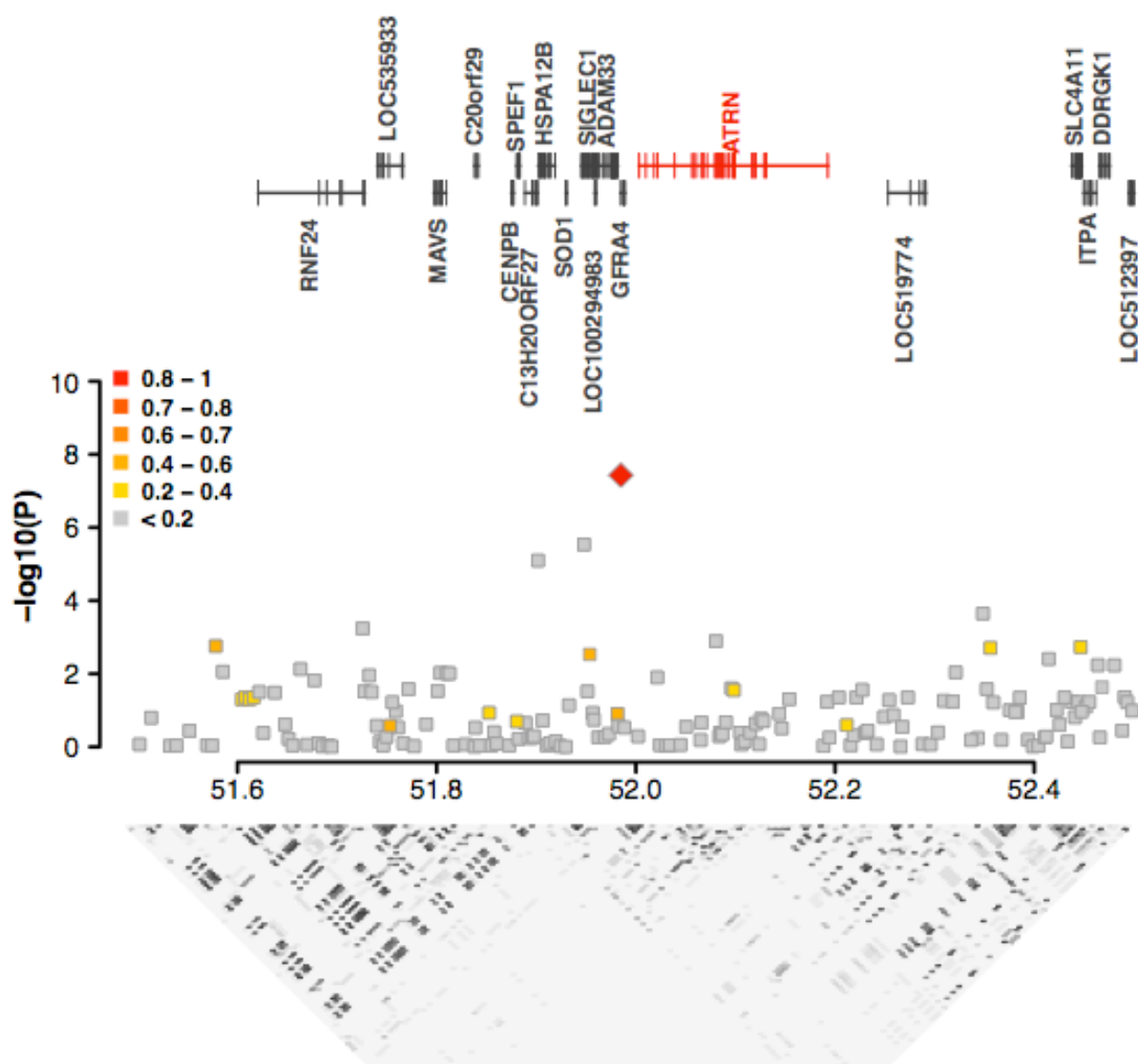

Supplement: Figure S9 — Detailed overview of the identified QTL region on BTA13 (51,500,000 bp–52,500,000 bp). The gene content was assessed based on the University of Maryland (UMD3.1) assembly of the bovine genome. The putative functional candidate gene is highlighted with red colour. The diamond represents the most significantly associated SNP while different colours represent the linkage disequilibrium (r2) between the most significantly associated SNP and all other SNPs within the displayed region. The heatmap displays the pairwise linkage disequilibrium. (PDF) [file pone.0036346.s009.pdf]

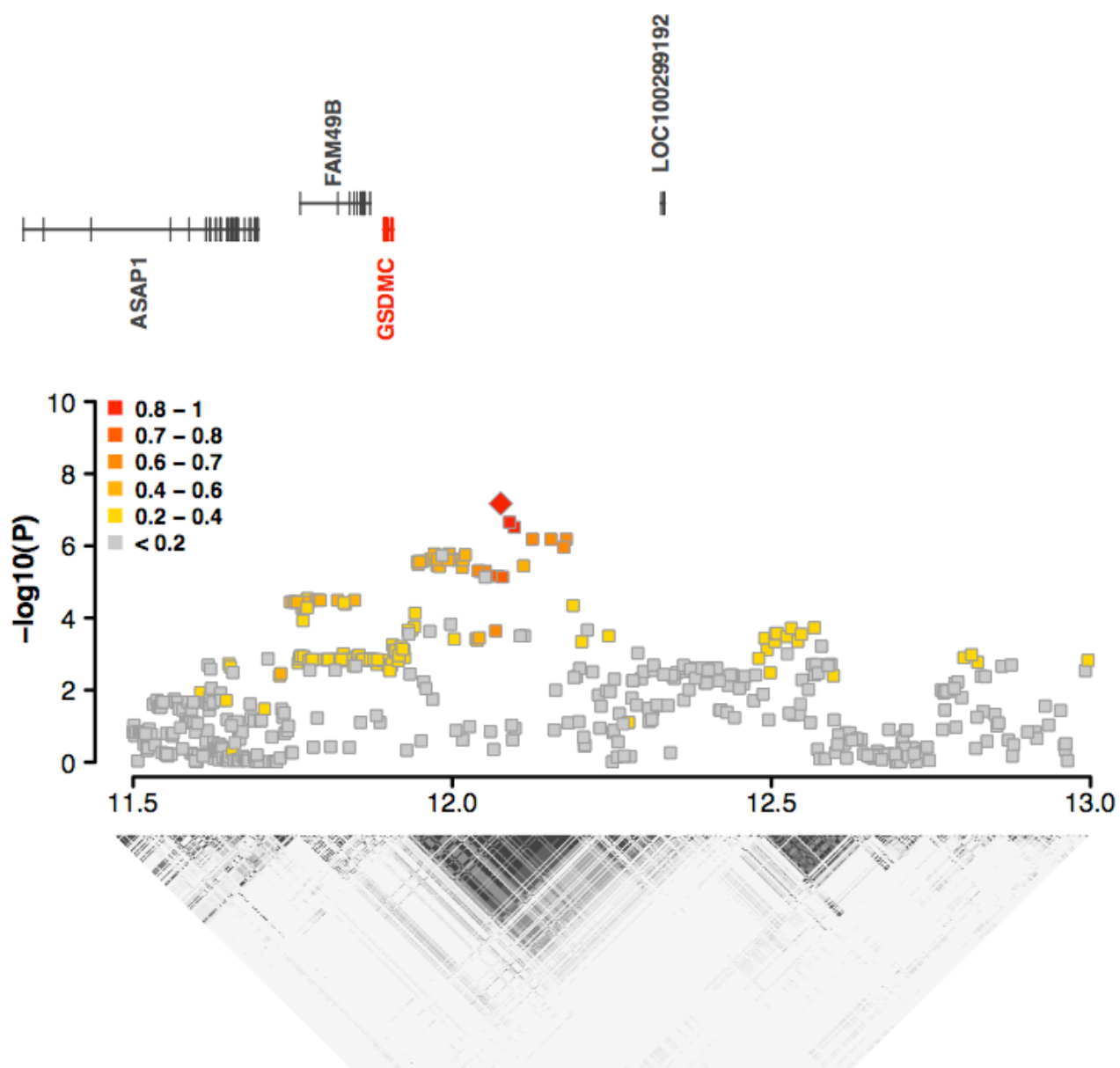

Supplement: Figure S10 — Detailed overview of the identified QTL region on BTA14 (11,500,000 bp–13,000,000 bp). The gene content was assessed based on the University of Maryland (UMD3.1) assembly of the bovine genome. The putative functional candidate gene is highlighted with red colour. The diamond represents the most significantly associated SNP while different colours represent the linkage disequilibrium (r2) between the most significantly associated SNP and all other SNPs within the displayed region. The heatmap displays the pairwise linkage disequilibrium. (PDF) [file pone.0036346.s010.pdf]

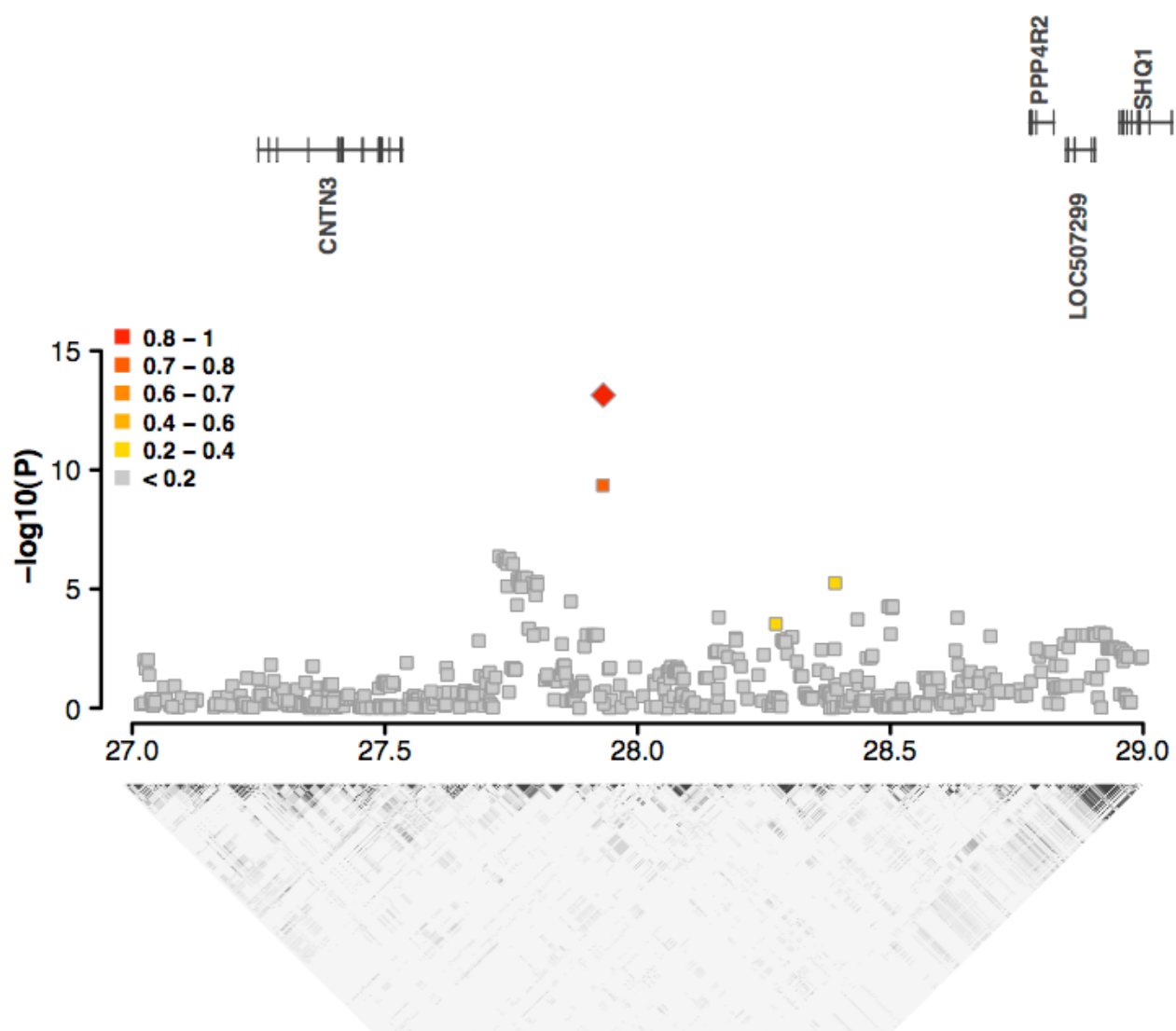

Supplement: Figure S11 — Detailed overview of the identified QTL region on BTA22 (27,000,000 bp–29,000,000 bp). The gene content was assessed based on the University of Maryland (UMD3.1) assembly of the bovine genome. The putative functional candidate gene is highlighted with red colour. The diamond represents the most significantly associated SNP while different colours represent the linkage disequilibrium (r2) between the most significantly associated SNP and all other SNPs within the displayed region. The heatmap displays the pairwise linkage disequilibrium. (PDF) [file pone.0036346.s011.pdf]

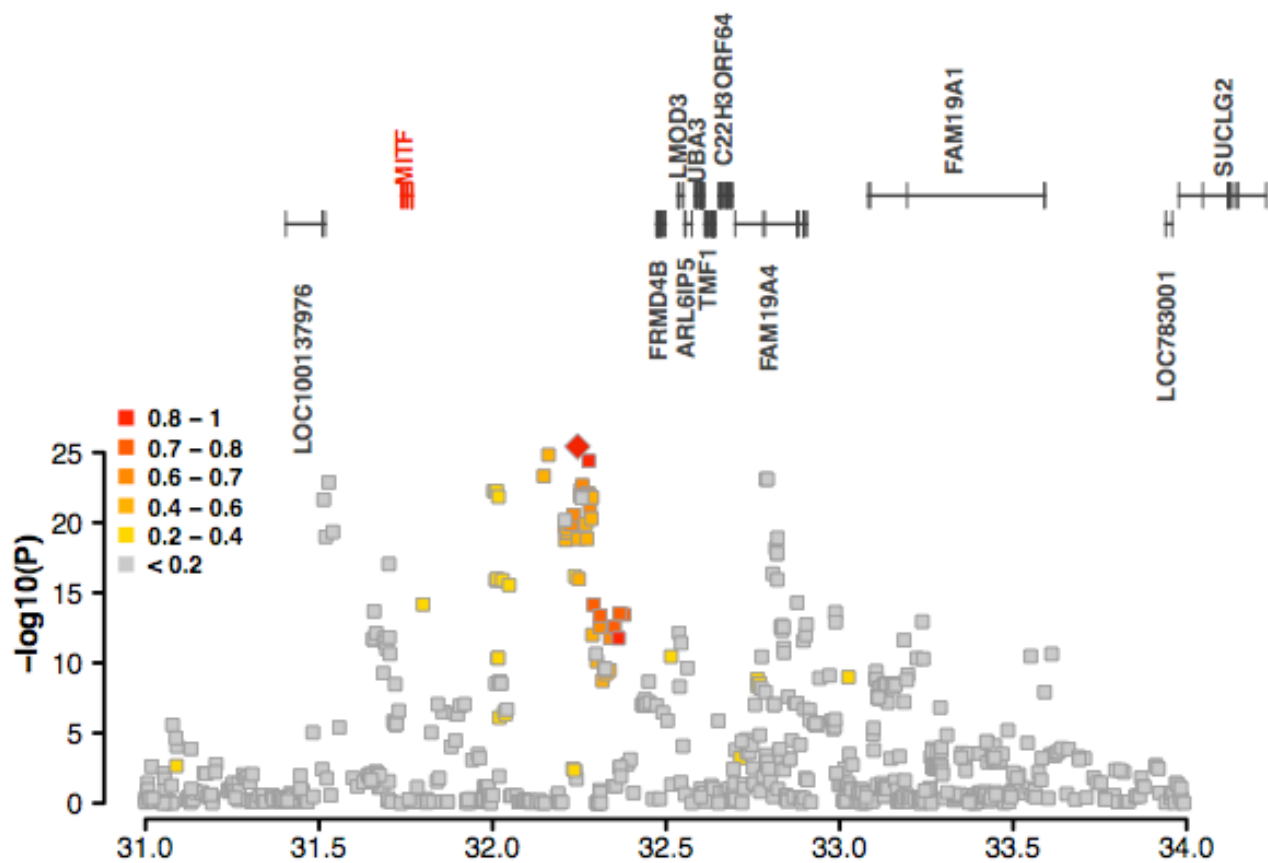

Supplement: Figure S12 — Detailed overview of the identified QTL region on BTA22 (31,000,000 bp–34,000,000 bp). The gene content was assessed based on the University of Maryland (UMD3.1) assembly of the bovine genome. The putative functional candidate gene is highlighted with red colour. The diamond represents the most significantly associated SNP while different colours represent the linkage disequilibrium (r2) between the most significantly associated SNP and all other SNPs within the displayed region. The heatmap displays the pairwise linkage disequilibrium. (PDF) [file pone.0036346.s012.pdf]

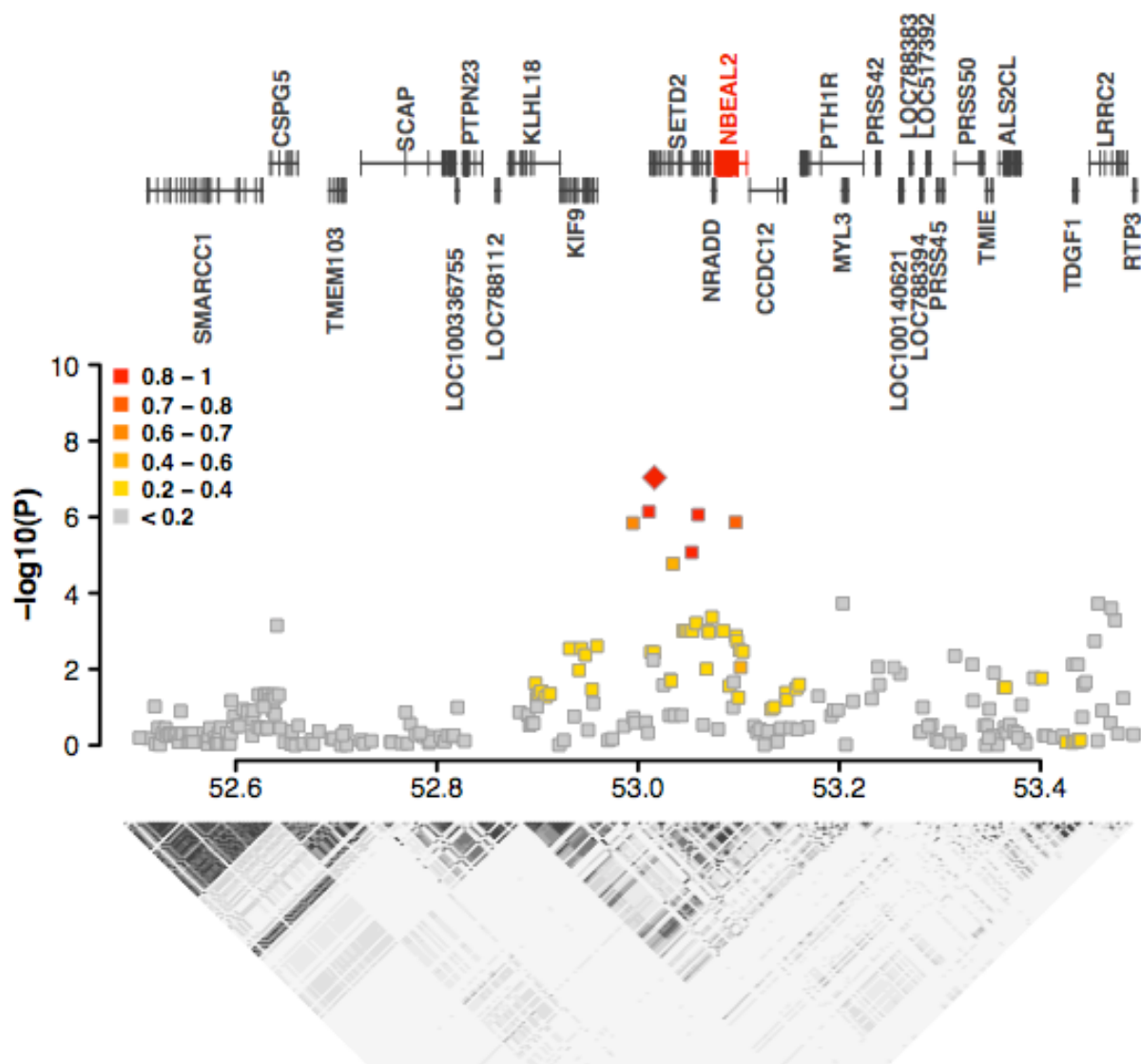

Supplement: Figure S13 — Detailed overview of the identified QTL region on BTA22 (52,500,000 bp–53,500,000 bp). The gene content was assessed based on the University of Maryland (UMD3.1) assembly of the bovine genome. The putative functional candidate gene is highlighted with red colour. The diamond represents the most significantly associated SNP while different colours represent the linkage disequilibrium (r2) between the most significantly associated SNP and all other SNPs within the displayed region. The heatmap displays the pairwise linkage disequilibrium. (PDF) [file pone.0036346.s013.pdf]

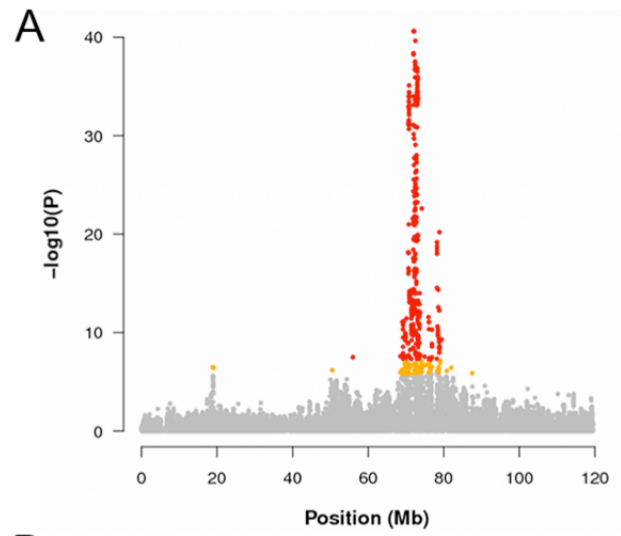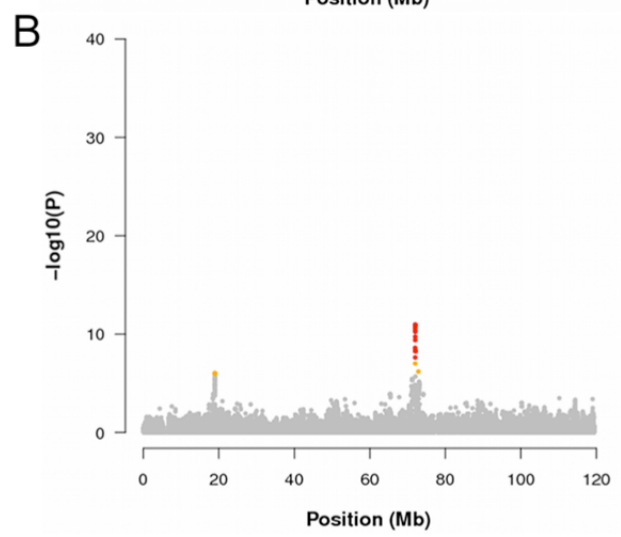

Supplement: Figure S14 — Association of 30,985 SNPs on Chromosome 6 with ambilateral circumocular pigmentation in 3579 animals of the Fleckvieh population. Results for the initial analysis (A) and for the analysis conditional on the BTB-00263209 SNP (B). Orange dots represent significantly associated SNPs on a chromosome-wide level, red dots represent significantly associated SNPs on a genome-wide level. (PDF) [file pone.0036346.s014.pdf]

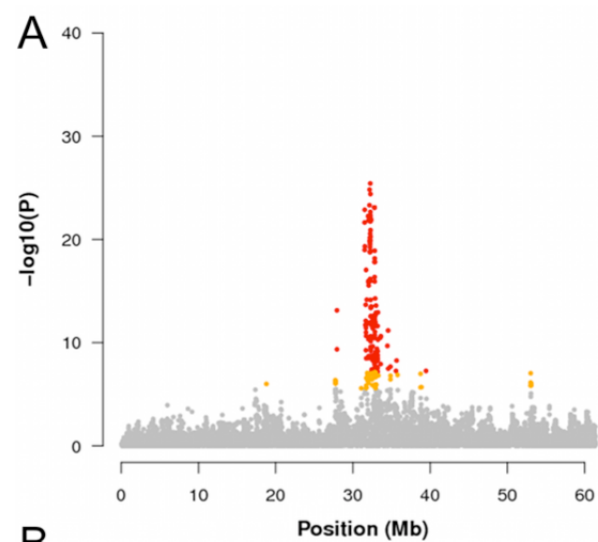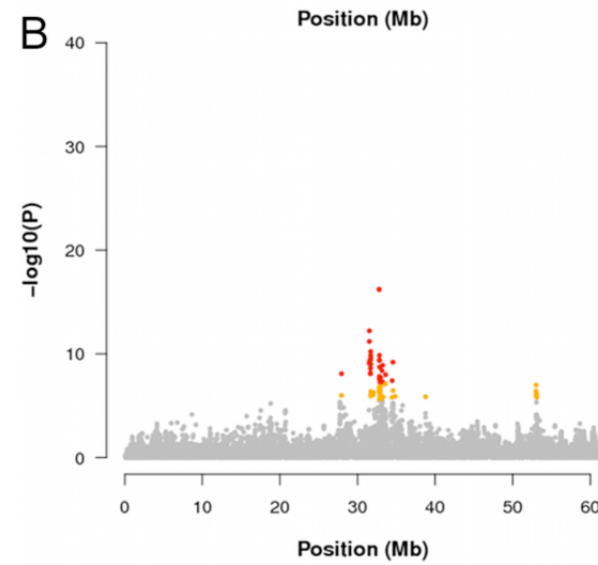

Supplement: Figure S15 — Association of 16,722 SNPs on Chromosome 22 with ambilateral circumocular pigmentation in 3579 animals of the Fleckvieh population. Results for the initial analysis (A) and for the analysis conditional on the BovineHD2200009208 SNP (B). Orange dots represent significantly associated SNPs on a chromosome-wide scale, red dots represent significantly associated SNPs on a genome-wide scale. (PDF) [file pone.0036346.s015.pdf]

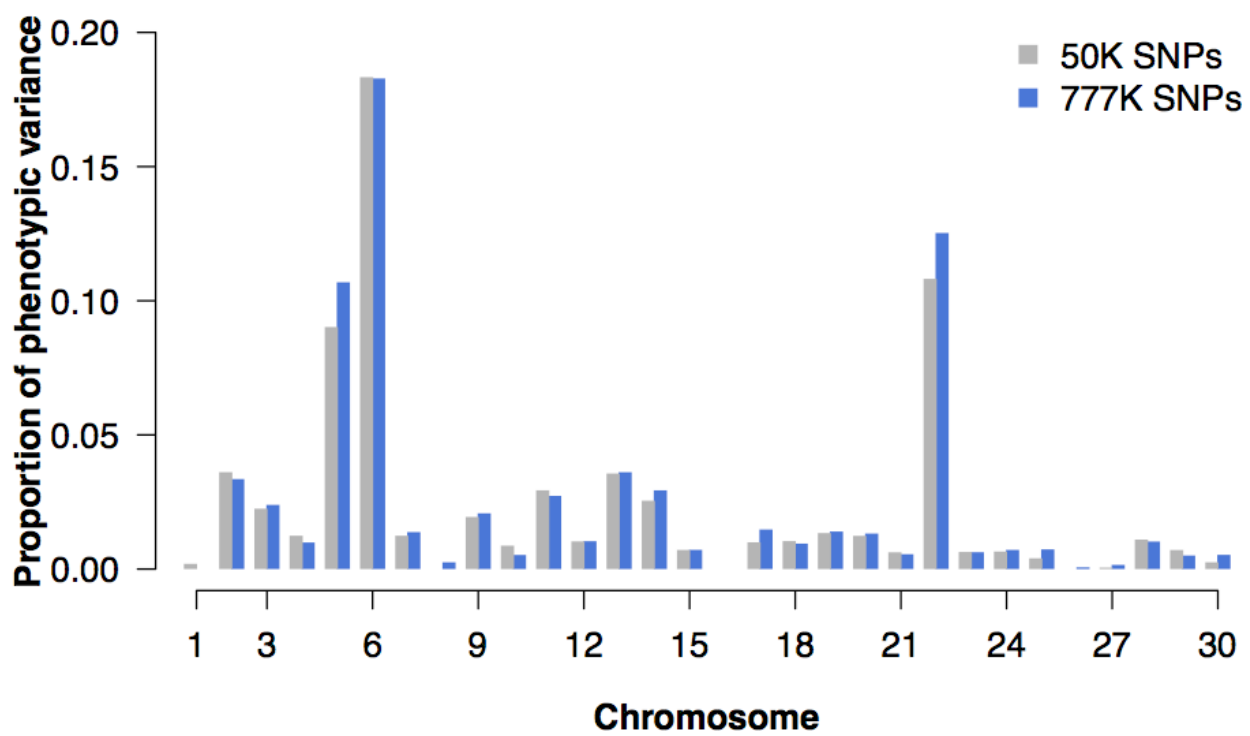

Supplement: Figure S16 — Effect of different marker densities on the chromosomal partitioning of the genetic variance. The grey and blue bars indicate the fraction of phenotypic variance attributed to a particular chromosome using the genomic relationship matrices built based on medium-density and high-density SNP information, respectively. (PDF) [file pone.0036346.s016.pdf]

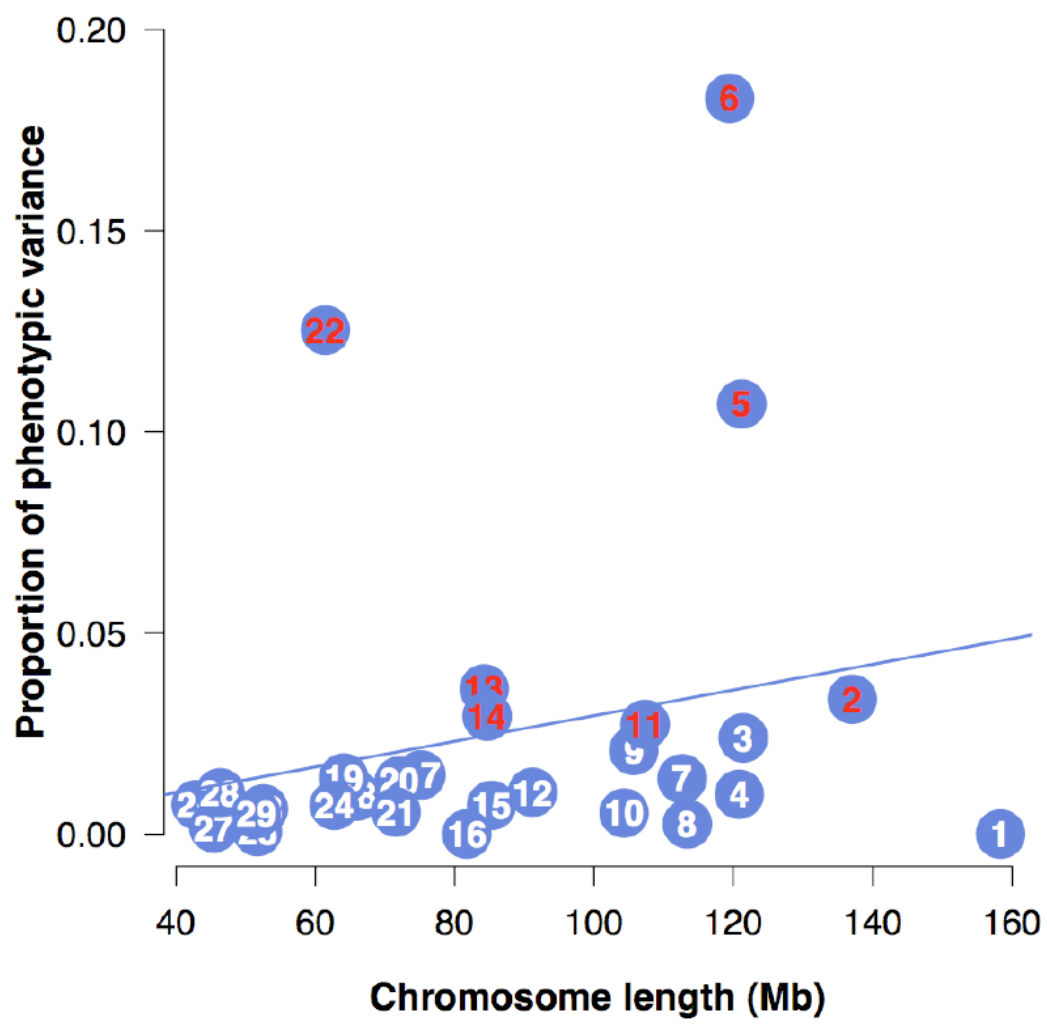

Supplement: Figure S17 — Correlation between chromosome length and the fraction of phenotypic variance explained. The estimate of the proportion of phenotypic variance explained by a particular chromosome is displayed as a function of the physical chromosome length (in Mb units). Red numbers indicate chromosomes with identified QTL. The blue line is a linear regression line with slope 3.2×10^−4^ (r2 = 0.06). (PDF) [file pone.0036346.s017.pdf]

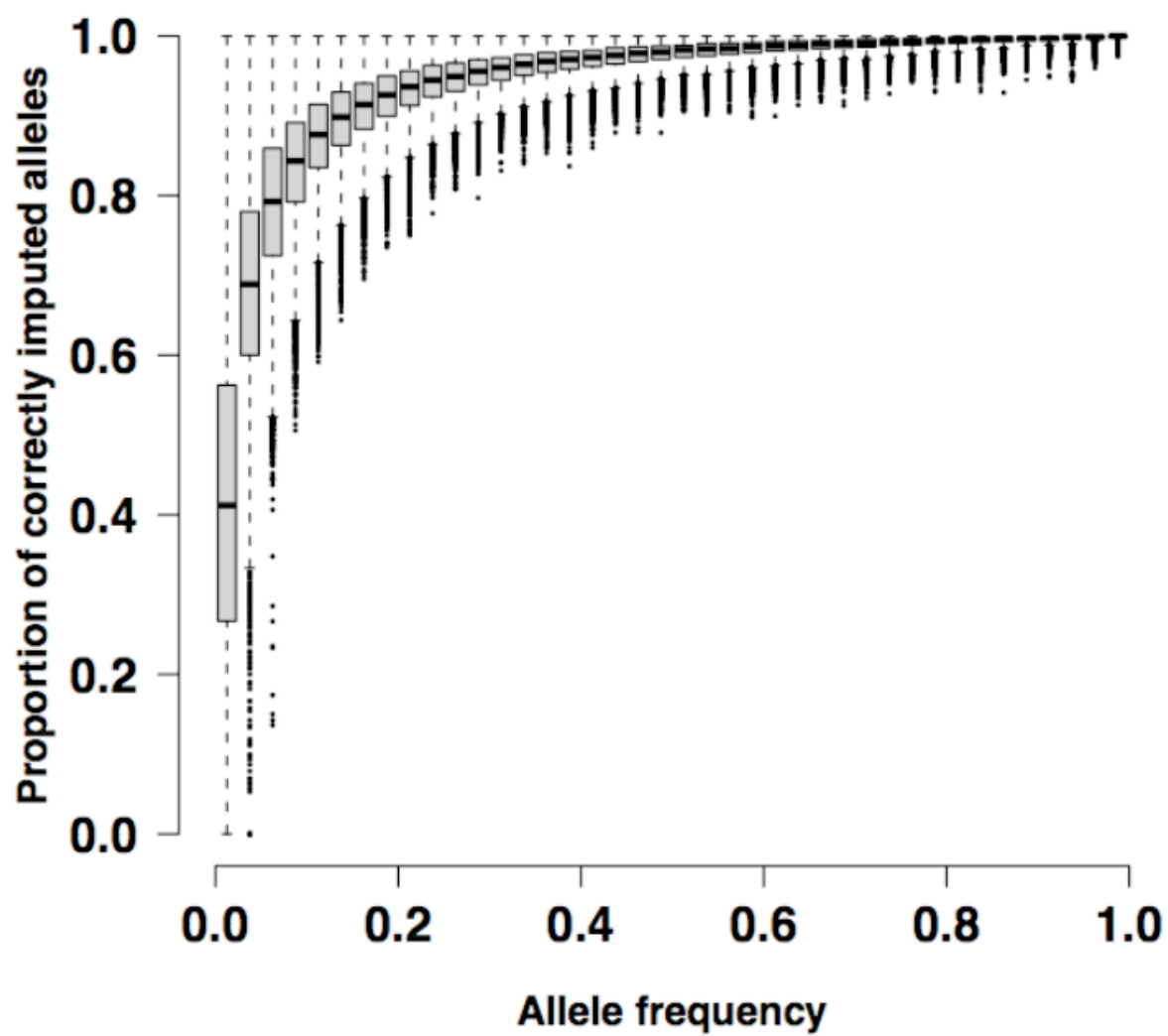

Supplement: Figure S18 — Accuracy of the imputed genotypes. Imputation accuracy was assessed based on genotypes of 613,232 chromosome-wide distributed SNPs of 402 animals. The proportion of correctly imputed alleles is displayed as a function of the allele frequency. The boxplots show the results for allele frequency bins of 2.5%. The concordance between imputed and true allele was poor (41.06%) for rare alleles (i.e. alleles with a frequency <2.5%), while imputation of frequent alleles (i.e. alleles with a frequency >65%) resulted in an allelic concordance >99%. (PDF) [file pone.0036346.s018.pdf]
