# Supplementary material for: Identification of QTL for UV-Protective Eye Area Pigmentation in Cattle by Progeny Phenotyping and Genome-Wide Association Analysis
Source: PLoS One. 2012 May 2;7(5):e36346. doi: 10.1371/journal.pone.0036346 (PMC3342244; doi:10.1371/journal.pone.0036346)
Supplement: Table S1 — Number of SNPs not passing the quality control parameters for the medium-density (54K v1 , 54K v1 ) and the high-density (777K) datasets. The number of SNPs and animals not passing the applied quality parameters as well as the final number of SNPs and animals is given for the two medium-density (54Kv1, 54Kv2) and for the high-density (777K) dataset, respectively (some SNPs failed for more than one quality control parameter). (PDF) [file pone.0036346.s019.pdf]

Quality parameter	54K*v1*	54K*v2*	777K
SNPs	54,001	54,609	777,962
Individuals	2,545	842	810
SNPs with unknown, Y-chromosomal or Mt-chromosomal position	549	1,075	3,302
SNPs with genotyping rate < 90%	761	256	7,854
SNPs with minor allele frequency < 0.5%	7,579	8,119	112,375
SNPs showing deviation from the Hardy-Weinberg Equilibrium (P<0.001)	704	401	4,578
Individuals with genotyping rate < 90%	6	11	1
Individuals with discrepancies between pedigree and genomic relationship	7	1	7
Duplicate SNPs (identical physical position, distinct SNP-id)	29	30	54
Remaining animals	2,532	830	802
Remaining SNPs	44,759	44,953	653,294
