# Supplementary material for: Identification of QTL for UV-Protective Eye Area Pigmentation in Cattle by Progeny Phenotyping and Genome-Wide Association Analysis
Source: PLoS One. 2012 May 2;7(5):e36346. doi: 10.1371/journal.pone.0036346 (PMC3342244; doi:10.1371/journal.pone.0036346)
Supplement: Table S2 — Number of SNPs for each of the three datasets after quality control. Numbers along the diagonal represent the final number of SNPs for the two medium-density (54Kv1, 54Kv2) and for the high-density (777K) dataset, respectively. Off-diagonal numbers indicate the intersection. (PDF) [file pone.0036346.s020.pdf]

| Dataset | 54K*v1* | 54K*v2* | 777K |
|---|---|---|---|
| 54K*v1* | 44,759 | 43,029 | 40,062 |
| 54K*v2* | | 44,953 | 40,350 |
| 777K | | | 653,294 |
