# Supplementary material for: Identification of QTL for UV-Protective Eye Area Pigmentation in Cattle by Progeny Phenotyping and Genome-Wide Association Analysis
Source: PLoS One. 2012 May 2;7(5):e36346. doi: 10.1371/journal.pone.0036346 (PMC3342244; doi:10.1371/journal.pone.0036346)
Supplement: Table S3 — Number of animals for each of the three datasets after quality control. Numbers along the diagonal represent the final number of SNPs for the two medium-density (54Kv1, 54Kv2) and for the high-density (777K) dataset, respectively. Off-diagonal numbers indicate the intersection. (PDF) [file pone.0036346.s021.pdf]

Dataset	54K*v1*	54K*v2*	777K
54K*v1*	2,532	-	521
54K*v2*		830	-
777K			802
