# Supplementary material for: Identification of QTL for UV-Protective Eye Area Pigmentation in Cattle by Progeny Phenotyping and Genome-Wide Association Analysis
Source: PLoS One. 2012 May 2;7(5):e36346. doi: 10.1371/journal.pone.0036346 (PMC3342244; doi:10.1371/journal.pone.0036346)
Supplement: Table S4 — Number of SNPs used for the evaluation of the imputation accuracy. (PDF) [file pone.0036346.s022.pdf]

| Chromosome | Chromosome length [Mb] | Number of high-density SNPs | Average distance between two high-density SNPs [bp] | Number of medium-density SNPs | Average distance between two medium-density SNPs [bp] |
|---|---|---|---|---|---|
| 1 | 158.34 | 40,100 | 3948 | 2607 | 60,665 |
| 2 | 137.06 | 33,962 | 4024 | 2070 | 65,977 |
| 3 | 121.43 | 30,848 | 3935 | 1907 | 63,520 |
| 4 | 120.83 | 30,482 | 3957 | 1936 | 62,235 |
| 5 | 121.19 | 29,777 | 4069 | 1648 | 73,515 |
| 6 | 119.46 | 30,781 | 3880 | 1969 | 60,474 |
| 7 | 112.64 | 28,290 | 3981 | 1751 | 64,219 |
| 8 | 113.38 | 24,935 | 4546 | 1812 | 62,346 |
| 9 | 105.71 | 26,663 | 3964 | 1578 | 66,877 |
| 10 | 104.3 | 27,127 | 3844 | 1652 | 62,440 |
| 11 | 107.31 | 28,544 | 3758 | 1691 | 63,419 |
| 12 | 91.16 | 22,267 | 4091 | 1280 | 71,106 |
| 13 | 84.24 | 18,409 | 4569 | 1372 | 61,149 |
| 14 | 84.65 | 19,369 | 4339 | 1418 | 58,682 |
| 15 | 85.3 | 21,860 | 3899 | 1267 | 66,809 |
| 16 | 81.72 | 21,055 | 3878 | 1249 | 65,104 |
| 17 | 75.15 | 19,808 | 3793 | 1223 | 61,280 |
| 18 | 66 | 17,556 | 3751 | 1008 | 64,947 |
| 19 | 64.06 | 17,289 | 3699 | 1058 | 60,115 |
| 20 | 72.04 | 19,426 | 3703 | 1193 | 60,022 |
| 21 | 71.6 | 18,336 | 3903 | 1056 | 67,461 |
| 22 | 61.44 | 16,605 | 3691 | 995 | 61,529 |
| 23 | 52.53 | 13,751 | 3815 | 811 | 64,475 |
| 24 | 62.71 | 15,987 | 3918 | 973 | 63,891 |
| 25 | 42.9 | 11,947 | 3581 | 743 | 57,610 |
| 26 | 51.68 | 13,932 | 3707 | 834 | 61,168 |
| 27 | 45.41 | 11,948 | 3800 | 735 | 61,761 |
| 28 | 46.31 | 11,832 | 3909 | 736 | 62,794 |
| 29 | 51.51 | 13,302 | 3864 | 800 | 63,958 |
| 30 | 148.82 | 17,106 | 8699 | 690 | 215,681 |
